# Identification of proteins and miRNAs that specifically bind an mRNA in vivo

**DOI:** 10.1038/s41467-019-12050-7

**Published:** 2019-09-16

**Authors:** Kathrin Theil, Koshi Imami, Nikolaus Rajewsky

**Affiliations:** 1 Systems Biology of Gene Regulatory Elements, Berlin Institute for Medical Systems Biology (BIMSB), Max Delbrück Center for Molecular Medicine in the Helmholtz Association (MDC), 13125 Berlin, Germany; 2 Proteome Dynamics, Max Delbrück Center for Molecular Medicine in the Helmholtz Association (MDC), 13125 Berlin, Germany; 3 Laboratory of Molecular and Cellular BioAnalysis, Graduate School of Pharmaceutical Sciences, Kyoto University, Kyoto, 606-8501 Japan

**Keywords:** Biochemistry, RNA, Biological techniques, Experimental organisms, Mass spectrometry

## Abstract

Understanding regulation of an mRNA requires knowledge of its regulators. However, methods for reliable de-novo identification of proteins binding to a particular RNA are scarce and were thus far only successfully applied to abundant noncoding RNAs in cell culture. Here, we present vIPR, an RNA-protein crosslink, RNA pulldown, and shotgun proteomics approach to identify proteins bound to selected mRNAs in *C. elegans*. Applying vIPR to the germline-specific transcript *gld-1* led to enrichment of known and novel interactors. By comparing enrichment upon *gld-1* and *lin-41* pulldown, we demonstrate that vIPR recovers both common and specific RNA-binding proteins, and we validate DAZ-1 as a specific *gld-1* regulator. Finally, combining vIPR with small RNA sequencing, we recover known and biologically important transcript-specific miRNA interactions, and we identify *miR-84* as a specific interactor of the *gld-1* transcript. We envision that vIPR will provide a platform for investigating RNA in vivo regulation in diverse biological systems.

## Abstract (web summary)

The entire mRNA interactome capture has been established but identification of specific mRNA-binding proteins has been challenging. Here, the authors developed an in vivo RNA–protein crosslinking and RNA pulldown approach and characterized novel interactors of the *C. elegans gld-1* mRNA, including DAZ-1 and *miR-84*.

## Introduction

Throughout their lives, mRNAs are bound by proteins which regulate their biogenesis, transport, stability, localization, and translation. Understanding how an mRNA is regulated requires knowledge of its complement of protein binders. Techniques for mRNA interactome capture allow identification of hundreds of RNA-binding proteins (RBPs) associated with poly(A)+ RNA in diverse biological systems (e.g., ^1–4^), but do not reveal the identity of specific mRNA-protein interactions. While the pool of RNAs bound by one RBP can be assessed by RBP immunoprecipitation (RIP) and crosslinking and immunoprecipitation (CLIP) approaches^5–7^, identifying all proteins bound to one particular RNA remains challenging. Conventional methods rely on genetic tagging of the RNA of interest or retrieval of in vitro-formed RNA-protein interactions (reviewed in ref. ^8^). Recently, also CRISPR-based approaches have been introduced to target native RNA-protein complexes^9,10^. These strategies have proven successful for several selected RNAs, but pose many problems impeding broader application. Constraints applying to one or all three approaches include: (1) not reflecting in vivo regulation, (2) laborious engineering, (3) high non-specific background, (4) non-physiological post-lysis associations of RNAs and proteins, and (5) inefficiency (reviewed in ref. ^8^). To overcome these limitations, additional methods have been developed in the last years. These employ crosslinking of native RNA-protein interactions in intact cells and pulldown of the RNA of interest by oligonucleotide probes under denaturing conditions^11–16^. This strategy enables efficient capture of endogenous transcripts with their native interactors while reducing background. However, these methods were not tested with mRNAs and key challenges remain. The lower the RNA of interest is expressed, the more input material is needed and the higher is the non-specific background from other abundant RNA-protein complexes. Consequently, most studies have focused on highly expressed transcripts thus far. Importantly, all these studies were performed in cell culture, not reflecting temporal and spatial regulation, e.g., interactions occurring in a specific developmental stage or in a specific tissue, altogether not recapitulating in vivo regulation.

Here, we describe vIPR (in vivo Interactions by ulldown of RNA), a method to identify factors binding to selected mRNAs in the complex context of an entire animal. We performed experiments in *C. elegans*. *C. elegans* is readily amenable to in vivo crosslinking of protein-RNA interactions by ultraviolet (UV) light^17–21^, and its germline is a well-established model for the study of post-transcriptional regulation (reviewed in ref. ^22^), with 3′ UTRs rather than promoters determining expression of most genes^23^. Applying vIPR, we not only identify protein binders of two mRNAs expressed in the *C. elegans* germline, but also recover microRNAs (miRNAs) binding differentially to them. Our method is not restricted to mRNAs, but can be applied to any similarly-expressed RNA molecule in *C. elegans*. We anticipate that our method can be extended to application in any organism amenable to in vivo crosslinking, thus shedding light on a multitude of in vivo regulatory mechanisms in diverse biological contexts.

## Results

### Development of vIPR

**Fig. 1:**
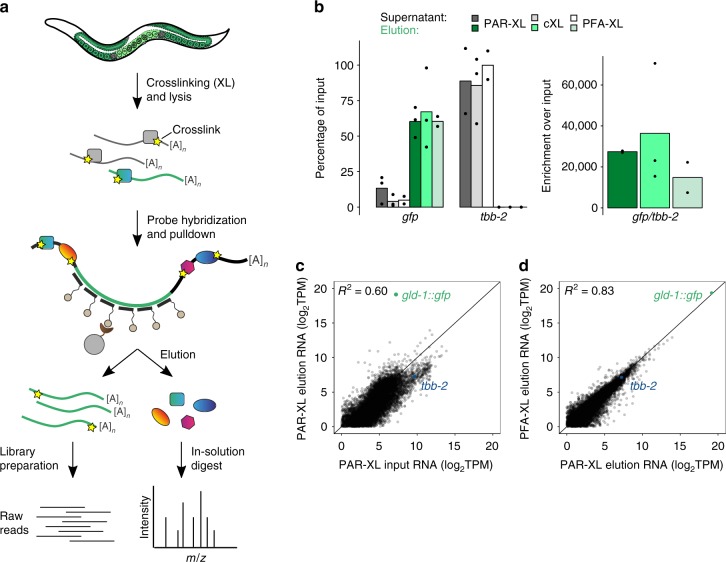
vIPR, an in vivo RNA-protein crosslinking and pulldown approach, efficiently and specifically enriches for RNA of interest. **a** Synchronized *C. elegans* young adults were crosslinked either by UV irradiation (PAR-XL, cXL) or chemically via paraformaldehyde (PFA-XL). After lysis, biotinylated 20 nt long DNA probes (here: complementary to the *gfp* coding sequence (green)) were added to hybridize to the RNA of interest. The probe-target complexes were captured by streptavidin-coated magnetic beads. After selective elution of protein or RNA, samples were subjected to mass spectrometry (LC–MS/MS) for protein identification or RNA sequencing/RT-qPCR for relative RNA quantification. *m*/*z*: mass-to-charge ratio. **b** vIPR leads to efficient and reproducible capture of the *gld-1::gfp* transcript, while *tbb-2*, an mRNA control, is depleted from pulldown elutions. RNA levels were measured from *n* = 3 (PAR-XL), *n* = 3 (cXL), *n* = 2 (PFA-XL) independent pulldown experiments by RT-qPCR, bars represent means. **c** RNA sequencing of input and pulldown elution samples confirms, transcriptome-wide, the specific enrichment of the *gld-1::gfp* transgenic RNA. Gene transcripts detected with a TPM count >1 in both samples are plotted. Input and elution samples are from independent PAR-XL pulldowns. Solid line represents diagonal. **d** RNA sequencing of pulldown elution samples employing either PAR-XL or PFA-XL shows reproducible RNA enrichment. Plotting analogous to **c**. PAR-XL, labeling of worms with 4-thiouridine (4SU) and crosslinking by UV 365 nm irradiation; cXL, crosslinking with UV 254 nm without prior labeling; PFA-XL, crosslinking with paraformaldehyde. TPM, transcripts per million. See Supplementary Data 1 for RNA sequencing transcript counts. Source data are provided as a Source Data file

To enable identification of proteins that interact with specific mRNAs in vivo, we developed vIPR (in vivo Interactions by Pulldown of RNA). vIPR relies on crosslinking of native protein–RNA interactions in live *C. elegans* with subsequent retrieval of the RNA of interest by an array of complementary oligonucleotides (Fig. 1a). To achieve efficient and specific capture of endogenous mRNA–protein complexes from *C. elegans*, we combined, modified, and optimized elements of methods recently applied to capture noncoding RNAs in cell culture (ChIRP-MS^11^; RAP-MS^12^). Compared to these studies, challenges were (1) the reduced crosslinking efficiency in vivo, (2) isolation of the mRNA from a complex mixture of tissues, and (3) the expected lower number of bound proteins due to comparatively short 3′ UTRs and thus limited space for specific protein binding.

As a proof-of-concept, we used a transgenic *C. elegans* strain expressing a GFP fusion protein of the RBP GLD-1 at endogenous levels^24^. GLD-1 binds its own transcript at five reproducible binding sites in the 3′ UTR^19^ and thus can serve as a positive control. Additionally, the nearly identical RBPs FBF-1 and FBF-2 (jointly called FBF) have been described to directly bind to *gld-1*^25,26^. To retrieve the *gld-1::gfp* transgenic transcript, we designed probes exclusively tiling the *gfp* coding sequence, reasoning that this would permit straightforward application of the method to any other *gfp* strain. After transcript capture, proteins and RNAs were selectively eluted by nuclease or protease treatment, respectively. Eluted proteins were then identified by quantitative mass spectrometry and RNA was assessed by RT-qPCR or RNA sequencing (Fig. 1a).

### Efficient and specific retrieval of the RNA of interest

To test which crosslinking method allows identification of specifically bound proteins in our in vivo setting, we performed pulldown experiments with three different crosslinking methods. First, we tested chemical crosslinking via paraformaldehyde (PFA-XL). PFA-XL leads to both nucleic acid–protein and protein–protein linkages. Second, we used UV light at 254 nm (cXL) which results in direct crosslinks between RNA and protein only. Third, we employed PAR-XL, labeling of nascent RNA with 4-thiouridine (4SU) and subsequent activation of these modified nucleotides by UV irradiation at 365 nm^19^, likewise yielding only direct RNA–protein interactions.

To assess *gld-1::gfp* pulldown efficiency and specificity, we measured RNA levels in input, supernatant, and elution samples by RT-qPCR. Irrespective of the crosslinking method, we retrieved ~60% of the input RNA (Fig. 1b, left). In contrast, an unrelated control transcript (*tbb-2*) was barely detected in the elution and consistently found not to be depleted from the supernatant. Compared to *tbb-2*, the *gld-1::gfp* transcript was enriched ~20,000-fold in elution samples (Fig. 1b, right).

To test whether the lysis conditions introduce a bias in terms of transcript retrieval, we compared transcript counts from RNA isolated after lysis during vIPR with RNA extracted from worms directly. The *gld-1::gfp* transcript was detected at similar relative counts in both samples (Supplementary Fig. 1a). In general, we found a high correlation of transcript counts between the two samples, indicating that pulldown lysis conditions do not significantly alter the relative copy numbers of transcripts (Supplementary Fig. 1a).

We also assessed *gld-1::gfp* enrichment transcriptome-wide by subjecting pulldown input and elution samples to RNA sequencing. We confirmed the specific enrichment of the *gld-1::gfp* mRNA and observed a high correlation between transcript abundances from pulldowns with different crosslinking methods (Fig. 1c, d; Supplementary Fig. 1b). Although ribosomal RNAs were massively depleted compared to input, we observed that absolute levels of the ribosomal 18S RNA were still high and comparable to the levels of *gld-1::gfp* in elution samples (Supplementary Fig. 1c).

To test whether the method can be readily applied to other, and more lowly expressed, *gfp* transcripts, we performed pulldowns in a strain expressing endogenously *gfp*-tagged *lin-41*, yielding similar transcript enrichment (Supplementary Fig. 1d–f). To further assess whether the method can be extended to target native transcripts of different abundances, we performed pulldowns for three endogenous, untagged transcripts (*gld-1*, *lin-41*, and *alg-1*; Supplementary Fig. 1g), each using 10 transcript-specific probes tiling the entire transcript rather than only the coding sequence (CDS). All pulldowns recovered ~70% of the respective target RNA (Supplementary Fig. 1h), with magnitudes of enrichment similar to the *gld-1::gfp* pulldown (Supplementary Fig. 1i–l). In summary, vIPR enables highly specific and efficient enrichment of an RNA of interest.

### Specific protein enrichment by vIPR with cXL

**Fig. 2:**
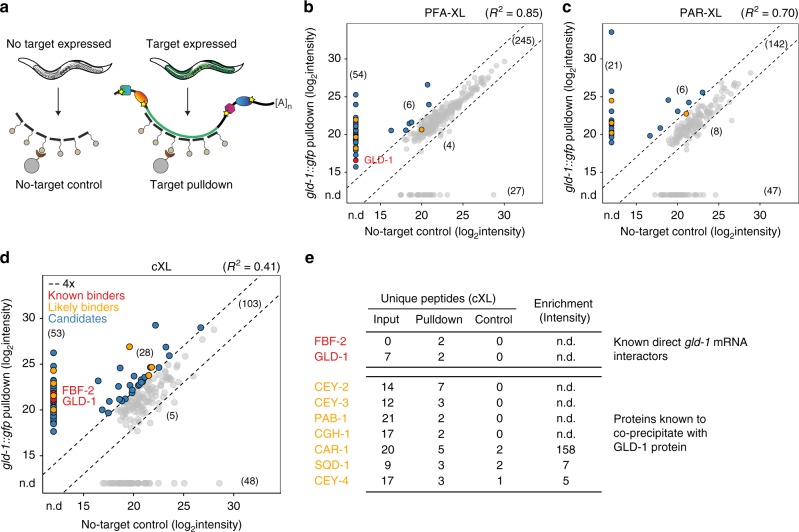
vIPR with cXL specifically enriches for known interactors of *gld-1* mRNA and GLD-1 protein. **a** To control for unspecific background, vIPR is performed in strains expressing and not expressing the *gfp*-tagged RNA of interest in an identical manner. **b**–**d** vIPR of *gld-1::gfp* with PFA-XL (**b**), PAR-XL (**c**), and cXL (**d**). Shown are peptide raw intensities from target pulldowns versus corresponding controls. *R*^2^ was calculated for proteins detected in both samples. Numbers in brackets indicate the protein identifications per category. Blue dots denote interaction candidates; red and orange dots denote known and likely interactors of *gld-1* mRNA (see **e**). n.d., not detected. **e** Proteins described to interact with either *gld-1* mRNA or GLD-1 protein were specifically recovered after pulldown of *gld-1::gfp*. Red indicates known *gld-1* mRNA regulators^19,25,26^, orange indicates proteins that were consistently found to co-precipitate with GLD-1 protein^32,33^. Shown are numbers of unique peptides identified in input, target pulldown and no-target control, as well as specific protein enrichment in pulldown versus no-target control, based on peptide intensities. See also Supplementary Data 2 for detected and enriched proteins

To compare the three crosslinking methods in terms of protein retrieval, we performed a pilot experiment testing all methods in parallel on the *gld-1::gfp* transcript. We devised a no-target control to assess protein background (Fig. 2a): For each crosslinking method, we performed the same procedure, using the same *gfp*-complementary probes, additionally on wild-type worms, which do not express the RNA of interest. This control accounts for all non-specific protein background resulting from direct binding to probes or beads, and indirect interactions mediated by RNA background.

While with all crosslinking methods, many proteins were detected non-specifically in both *gld-1::gfp* and no-target pulldown, vIPR with cXL led to the highest number of specifically enriched proteins in the target pulldown (Fig. 2b–d). To test whether the crosslinking method impacts protein detection, we subjected input samples from pulldowns with different crosslinking methods to mass spectrometry. The peptide intensities were generally comparable between different inputs (Supplementary Fig. 2a) and also reproduced in supernatant samples after pulldown (Supplementary Fig. 2b). Importantly, vIPR with cXL not only yielded the highest number of enriched proteins, but these also included both GLD-1 and FBF, the only *gld-1* regulators for which direct binding has been established to date^19,25,26^. These were absent from the control, and notably, FBF proteins were not detected in the corresponding input sample, suggesting low FBF protein levels and high sensitivity of our approach (Fig. 2e). A number of other proteins were suggested to be involved in post-transcriptional regulation of *gld-1*: The cytoplasmic poly(A) polymerases GLD-2 and GLD-4 have been reported to activate and/or stabilize the *gld-1* mRNA, likely by poly(A) tail extension^27,28^. These enzymes lack RNA-binding domains and are thought to be recruited to their targets by accessory proteins^29^. While GLD-3, RNP-8 and FBF have been found to co-precipitate with one or both enzymes in a complex with *gld-1* mRNA^27,28,30,31^, it is currently unclear which protein(s) mediate the specific RNA contact, and apart from FBF, none of them was detected in our pilot experiment.

Two independent studies reported sets of proteins that co-precipitate with GLD-1 protein^32,33^. Since many of these are known or predicted to bind RNA, it is possible that GLD-1 interacts with them via jointly bound RNA. We thus considered these proteins as additional candidate interactors of the *gld-1* transcript. Interestingly, all proteins (7/7) consistently identified in both studies were enriched in the *gld-1::gfp* cXL pulldown (Fig. 2e). Taken together, identification of known and anticipated binders of the *gld-1* transcript by vIPR with cXL suggests that the method enables discovery of transcript-specific RBPs.

### The *gld-1* mRNA is bound by RBPs that interact functionally

**Fig. 3:**
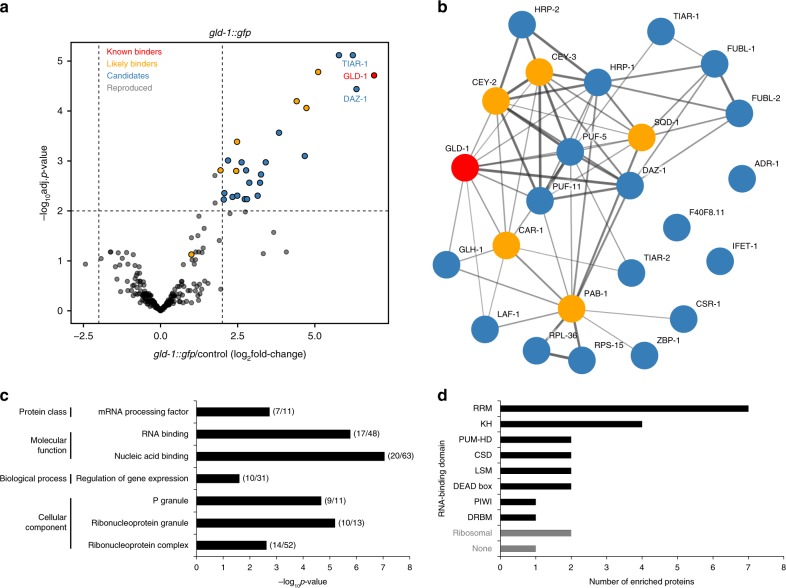
vIPR of *gld-1::gfp* reproducibly enriches for RBPs that are functionally linked. **a** Triplicate vIPR of *gld-1::gfp* identifies proteins that reproducibly enrich versus no-target control. Proteins reproducibly detected: *n* = 273, proteins reproducibly enriched: *n* = 25 (24 endogenous *C. elegans* proteins plus GFP). Mean fold-changes and *p*-values (moderated *t*-test; BH-corrected; Supplementary Data 3) from three independent pulldown experiments are plotted. Significance cut-off: adjusted *p*-value < 0.01, enrichment cut-off: >4-fold. **b** The reproducibly enriched proteins form a functional network. Confidence interaction map generated with string-db.org^35^. Thickness of edges represents degree of confidence of functional linkages. The number of edges is significantly higher than expected, with a *p*-value < 1 × 10^−16^. See also Supplementary Fig. 3b. Colors analogous to **a**. **c** Top over-represented gene ontology (GO) terms of reproducibly enriched proteins. Enrichment cut-off: >3-fold, significance (binomial test) cut-off: Bonferroni-corrected *p*-value < 0.01. Number of analyzed genes – background: 269 proteins with annotated GO terms, and consistently detected in all three *gld-1::gfp* pulldown replicates; test set: 24 enriched proteins (see **a**). Numbers of genes in test and background set per category are given in brackets next to corresponding bars. See also Supplementary Data 4. **d** Most enriched proteins are known or predicted to bind RNA. Given are the numbers of proteins with respective RNA-binding domains according to annotation from uniprot.org (April 2018)

In our pilot experiment, many of the enriched proteins were detected with few peptides only (Fig. 2e). To assess reproducibility of protein enrichment and to identify high-confidence in vivo interactors, we performed triplicate cXL vIPR pulldowns of the *gld-1::gfp* transcript. We employed label-free quantification (LFQ)^34^ to accurately determine peptide intensities and only considered proteins quantified in all three *gld-1::gfp* pulldowns. Of the 273 reproducibly detected proteins, 29 proteins were found >4-fold enriched in all replicates (Supplementary Fig. 3a). We determined significantly enriched proteins (significance cut-off: *p* < 0.01; moderated *t*-test; Benjamini-Hochberg (BH) correction), comparing all target pulldowns with all no-target controls (Fig. 3a). We could reproduce enrichment of most of the previously-identified known or likely binders of the *gld-1* mRNA (red and yellow dots, respectively) and additionally identified further candidates (blue dots).

We asked whether the identified proteins are functionally linked and/or share any characteristics, in support of authentic and direct interactors. We performed STRING network analysis^35^ and found that the set of candidate binders contains many more interaction edges than expected by chance (*p*-value < 1 × 10^−16^; Fig. 3b). To exclude that this is a general feature inherent to the pulldown procedure, we also tested sets of the same size randomly drawn from the non-enriched proteins in the pulldown sample. These sets displayed much less high-confidence interactions than the set of candidates (Supplementary Fig. 3b).

We also analyzed gene ontology (GO) terms of the candidates, using all reproducibly detected proteins as the background set. GO terms related to RNA metabolism were highly over-represented in the candidate set (Fig. 3c). Interestingly, many candidates (*n* = 10) were annotated to function in or associate with RNA granules, suggesting a role for these proteins in RNA storage. Crosslinking with cXL is assumed only to capture direct RNA-protein contacts. Consistently, most candidates (21/24) are described or predicted to have RNA-binding activity, with motifs belonging to diverse RNA-binding domain classes (Fig. 3d).

### vIPR allows identification of common and specific binders

**Fig. 4:**
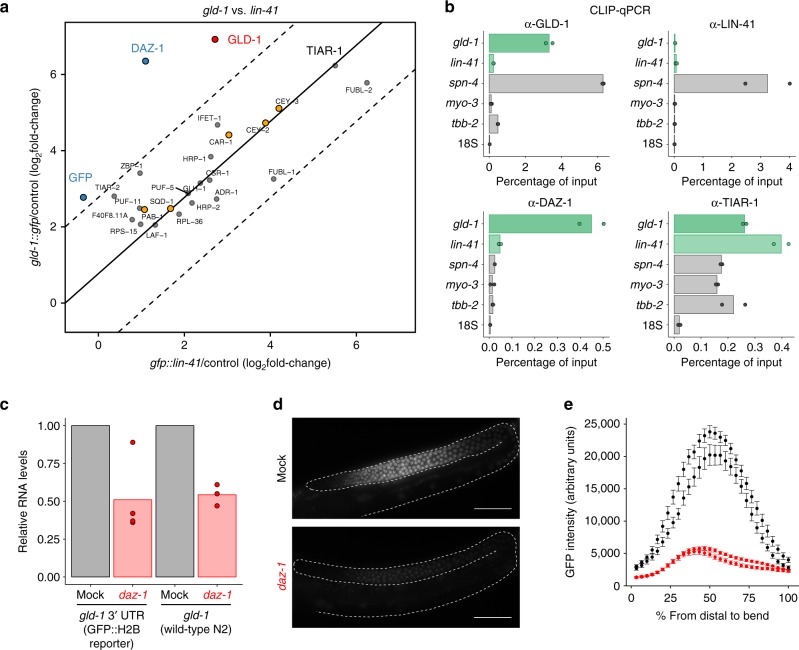
vIPR reveals common and specific RBPs, and identifies DAZ-1 as a *gld-1* regulator. **a** Comparison of protein enrichment from vIPR of the *gld-1::gfp* and *gfp::lin-41* transcripts. Proteins significantly more abundant for one transcript are colored blue (differential binding cut-off: >4-fold, adjusted *p*-value < 0.01, moderated *t*-test; BH-corrected; Supplementary Data 3). Red and yellow indicate known and likely binders of the *gld-1::gfp* transcript, respectively (see Fig. 2e). Solid line demarcates the transcript expression-adjusted diagonal (intercept = log_2_(250 TPM/147 TPM)), dashed lines demarcate the fold-change cut-offs. **b** Crosslinking and immunoprecipitation (CLIP) confirms that DAZ-1 specifically interacts with *gld-1* mRNA, while TIAR-1 binds all tested mRNAs. Experiments were performed with *C. elegans* strains expressing tagged versions of the indicated proteins. GLD-1 and LIN-41 CLIPs were performed as positive and negative controls for *gld-1* binding, respectively. The *spn-4* mRNA is a known germline target of LIN-41^77^ and served to control for the integrity of tagged LIN-41 protein. Control CLIPs in wild-type worms did not enrich for any of the tested transcripts (<0.02% of input detected after CLIP). Experiments were performed with *n* = 2 biological replicates, bars represent means. Green: mRNAs assessed by vIPR. **c** Relative RNA levels of *gld-1* 3′ UTR reporter in transgenic worms, and endogenous *gld-1* in N2 worms after mock and *daz-1* RNAi treatment, measured by Nanostring nCounter and normalized to *tbb-1/tbb-2* levels. Bars represent means, data are from *n* = 4 (*gld-1* reporter) and *n* = 3 (wild-type N2) independent biological replicates. See also raw data in Supplementary Data 5. **d** Representative microscope images of *gld-1* 3′ UTR reporter fluorescence in gonads of transgenic worms upon RNAi treatment. Dashed lines encircle gonads. Scale bar: 50 µm. **e** Quantification of GFP fluorescence along the distal gonad. Plotted are mean GFP intensities for two independent transgenic lines of the *gld-1* 3′ UTR reporter upon mock and *daz-1* RNAi treatment. Number of analyzed gonads – mock: *n* = 24, *n* = 18; *daz-1*: *n* = 35, *n* = 23. Error bars represent SE. Source data are provided as a Source Data file

Many RBPs are known to be promiscuous binders. We aimed to identify candidates for specific regulation of *gld-1*. To this end, we performed additional vIPR experiments with another mRNA, *gfp::lin-41*. In adult *C. elegans*, *lin-41* is predominantly expressed in the germline, as is *gld-1*. Using the same *gfp-*complementary probes as for *gld-1::gfp*, we performed triplicate pulldowns and reproducibly detected 278 proteins. We found 15 proteins consistently enriched >4-fold (Supplementary Fig. 4a) and 9 proteins that passed our significance cut-off (Supplementary Fig. 4b). We compared transcript expression-adjusted fold-changes of the *gld-1::gfp* interactome with the interactome of *gfp::lin-41*. Many proteins were identified in both pulldowns at similar levels, suggesting factors involved in general mRNA processing or shared regulation of both transcripts. While no proteins were found to be significantly more abundant in *gfp::lin-41* pulldowns (cut-offs: enrichment >4-fold, adjusted *p*-value < 0.01), the *gld-1::gfp* transcript consistently enriched for the proteins GLD-1 and DAZ-1 (Fig. 4a).

The preferential enrichment of GLD-1 in *gld-1::gfp* pulldowns is consistent with the *gld-1* transcript being one of GLD-1 protein’s top targets, whereas *lin-41* has not been described to be bound by GLD-1^19^. Little is known about the regulation of *lin-41* mRNA except for its interaction with the *let-7* miRNA which is critical for *C. elegans* development^36–39^. We did not detect ALG-1, the miRNA effector protein in *C. elegans*, prompting the question whether the *lin-41* 3′ UTR is efficiently captured. Since we used probes solely annealing to the *gfp* coding sequence, we asked whether the pulldown procedure might deplete for transcript regions not covered by probes, e.g., due to shearing or partial RNA degradation. We compared RNA retrieval for different regions of the *gfp::lin-41* transcript by RT-qPCR. For all regions, ~60–80% of input RNA was recovered, indicating that transcript regions not covered by probes were still efficiently captured (Supplementary Fig. 4c). Taken together, the comparison of *gfp*-tagged *gld-1* and *lin-41* pulldowns suggests that vIPR allows identification of both promiscuous and specific RBPs.

### vIPR of endogenous transcripts

Using transgenic strains together with probes directed against the transgenic sequence provides the advantage of accounting for both general and probe-specific background, and facilitates comparison of binders between transcripts. While CRISPR systems enable facile editing of endogenous loci, insertion of a heterologous sequence bears the risk of losing or gaining interactions by destruction or introduction of regulatory sequences.

We showed that endogenous transcripts can be captured efficiently and specifically by transcript-specific probes (Supplementary Fig. 1h–l). To compare proteins interacting with endogenous and transgenic transcripts, we performed two pulldowns for the endogenous *gld-1* transcript and one additional vIPR experiment for *gld-1::gfp*. We again controlled for background binding by pulldown with *gfp*-complementary probes in wild-type worms. We found 23 proteins to be consistently enriched >4-fold in all three pulldowns compared to respective controls (Supplementary Fig. 4d), and 17 that passed our significance cut-off (Supplementary Fig. 4e). Of the 24 candidates identified previously for *gld-1::gfp* (Fig. 3a), we reproducibly detected 18 in our new analysis, with 13 again passing our stringent cut-offs (Supplementary Fig. 4e). The reproducible enrichment of proteins in vIPR of both endogenous and transgenic *gld-1* argues against major differences in protein retrieval between transgenic and endogenous, untagged transcripts.

We also performed additional vIPR experiments for *lin-41* and reproducibly identified 5 of the 9 previously identified candidates, with 2 of them passing our stringent cut-offs (Supplementary Fig. 4f). Importantly, all the previously identified candidates were more abundant in the *lin-41* pulldowns compared to the controls. Notably, the two proteins additionally identified as significantly enriched, GLH-1 and H05C05.1, were also found enriched in our previous analysis of *gfp::lin-41*, but did not pass our cut-offs.

Both *gld-1::gfp* and *gfp::lin-41* are amongst the top 15% of protein-coding transcripts (Supplementary Fig. 1d, e). To assess the limits of the applicability of vIPR, we assessed captured proteins of the lowly expressed *alg-1* transcript. While several of the RBPs identified in previous vIPR experiments were also identified in *alg-1* pulldowns, they did not pass our enrichment cut-offs, and did not separate from other reproducibly-detected proteins with no described RNA-binding activity (Supplementary Fig. 4g).

Taken together, vIPR enables identification of protein interactors of both transgenic and endogenous transcripts. The lack of significantly enriched proteins after *alg-1* pulldown indicates, however, that the method in its current state does not enable reliable discrimination of specific binders from noise for lowly expressed transcripts.

### DAZ-1 binds and regulates the *gld-1* mRNA

To validate the identified interactions for *gld-1* and *lin-41*, we performed independent CLIP-qPCR experiments for two candidates: DAZ-1, an RBP expected to selectively bind *gld-1*, and TIAR-1, a protein expected to bind both *gld-1* and *lin-41* mRNAs. As positive and negative controls, we tested GLD-1 and LIN-41. GLD-1 CLIP confirms the preferential binding of *gld-1* mRNA, whereas CLIP of LIN-41 suggests that it neither binds *gld-1* nor *lin-41* (Fig. 4b). Consistent with the pulldown results, TIAR-1 promiscuously binds to many mRNAs, whereas DAZ-1 preferentially binds *gld-1*.

DAZ-1 homologs have been described to stabilize target transcripts and/or activate their translation^40^. To explore whether DAZ-1 could function similarly in regulation of the *gld-1* transcript, we generated two independent lines of a single-copy *C. elegans* reporter strain, expressing the *gld-1* 3′ UTR fused to the GFP::H2B CDS in the germline. We first compared the levels of both endogenous *gld-1* mRNA and the *gld-1* 3′ UTR reporter between mock and *daz-1* RNAi treatment. Knockdown of *daz-1* resulted in reduction of both endogenous *gld-1* and *gld-1* reporter levels by ~50%, consistent with a role of DAZ-1 in *gld-1* transcript stabilization (Fig. 4c, Supplementary Fig. 4h). To assess spatial reporter protein expression, we recorded GFP fluorescence in worm gonads (Fig. 4d). Quantification of mean GFP intensities along the distal gonad, the area of DAZ-1 expression^41^, revealed a drastic drop in reporter protein levels to <25% upon *daz-1* knockdown (Fig. 4e). This drop exceeds the changes observed on RNA level, supporting the additional suggested function of DAZ-1 as a translational activator^42^. In conclusion, we validated the binding of TIAR-1 to both *gld-1* and *lin-41* and provide evidence that DAZ-1 is a specific positive regulator of *gld-1* expression.

### Transcript-specific enrichment of miRNAs

We did not detect ALG-1, the microRNA effector protein, in any of our vIPR experiments. However, *lin-41* and *alg-1* are well-established miRNA targets^17,18,21,38,39,43^. Transcript-specific miRNAs can in principle be recovered by probe-mediated RNA pulldown^44^. We asked whether vIPR allows, in addition to identification of in vivo protein binders, discovery of transcript-specific miRNAs. To this end, we performed small RNA sequencing on pulldown samples, and compared miRNA counts with corresponding no-target controls.

**Fig. 5:**
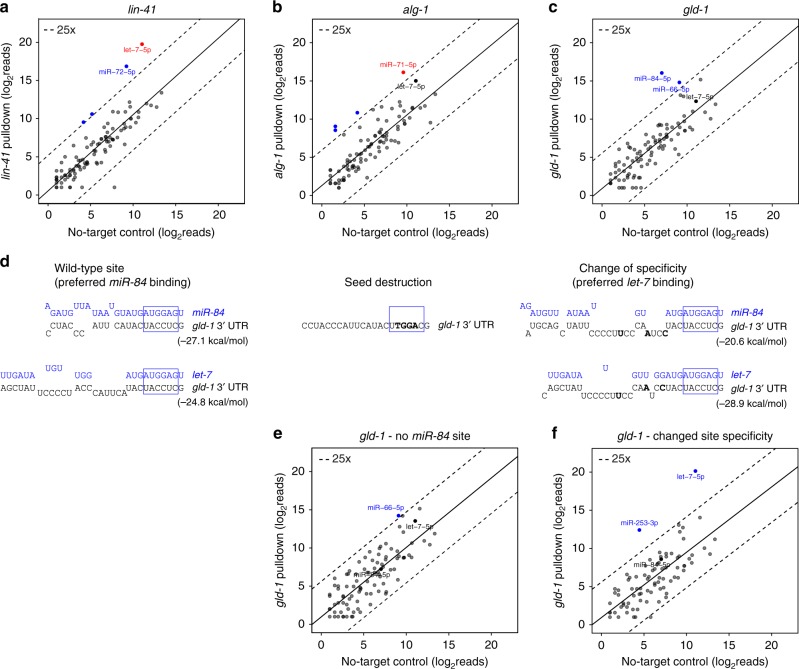
vIPR allows identification of transcript-specific miRNAs. **a–c** vIPR identifies miRNAs that specifically enrich versus no-target control (blue dots) for endogenous *lin-41* (**a**), *alg-1* (**b**), and *gld-1* (**c**) transcripts. Known interactions are colored in red. Plotted are miRNAs found in both target and no-target pulldown. Solid line: linear regression of log_2_-transformed reads. **d** Base-pairing predictions (RNAhybrid^45^; version 2.2) for the *miR-84* and *let-7* miRNAs with the *gld-1* 3′ UTR. Edits introduced by CRISPR to either abrogate *miR-84* binding (seed destruction) or to convert the *miR-84* site into a *let-7* binding site (change of specificity) are shown as bold nucleotides. The blue boxes highlight the miRNA seed. **e** Editing of the *let-7* family seed complement leads to abrogation of *miR-84* binding. **f** Editing of seed-distal bases results in abrogation of *miR-84* binding and enrichment of the *let-7* miRNA upon *gld-1* pulldown. For each target, one replicate of two independent pulldown experiments is shown. See also Supplementary Data 6 for miRNA reads

Importantly, *let-7* was highly enriched in both the endogenous *lin-41* and *gfp::lin-41* pulldowns (Fig. 5a, Supplementary Fig. 5a), consistent with its role as a known binder and crucial regulator of developmental timing^36,38,39^. Although the *alg-1* transcript is expressed at much lower levels (Supplementary Fig. 1g), we recovered its interaction with *miR-71* (Fig. 5b), that was shown to reduce ALG-1 levels during aging^17,18,43^.

Performing pulldowns for endogenous and transgenic *gld-1*, we identified *miR-84* to be specifically and highly enriched (Fig. 5c, Supplementary Fig. 5b). Independent evidence for this interaction comes from miRNA:target chimeric reads derived from ALG-1 iCLIP experiments^17^. In additional support, mining of the *gld-1* 3′ UTR for predicted hybrids^45^ with *miR-84* yielded a highly stable structure with a sequence stretch that is consistent with the chimera data (Fig. 5d).

Intriguingly, *let-7* and *miR-84* belong to the same miRNA family and thus share the same seed sequence (Supplementary Fig. 5c). Despite the seed generally being the predominant determinant of a miRNA’s pool of targets, it has been reported that miRNA family members can target specific subsets of transcripts, which depends on differential base pairing outside the seed^17,46^. This has been shown to be particularly prevalent for the *let-7* family^17^. The predicted *miR-84* site in the *gld-1* 3′ UTR exhibits extended seed pairing with both *let-7* and *miR-84* (Fig. 5d). However, pulldown of *gld-1* specifically enriched for *miR-84* (Fig. 5c, Supplementary Fig. 5b). This is consistent with the higher predicted stability of the hybrid and the higher number of retrieved chimeric reads for the *miR-84:gld-1* interaction (Fig. 5d, Supplementary Fig. 5d).

To confirm that *miR-84* binds *gld-1*, we CRISPR-edited four bases in the seed-complementary region of the endogenous locus (Fig. 5d). Abolishing the predicted seed pairing led to loss of *miR-84* enrichment (Fig. 5e). To test whether a switch in site specificity can be recovered by vIPR, we additionally generated a CRISPR-edited strain that harbors three base substitutions outside of the seed-complementary region (Fig. 5d). The edit effectively converted the *miR-84* site to a *let-7* site, with the *gld-1* pulldown now yielding *let-7* as the most enriched miRNA (Fig. 5f).

Taken together, we recovered known and biologically important miRNA:target interactions, and we identified *miR-84* as a specific binder of the *gld-1* transcript. We conclude that vIPR enables differential identification of transcript-specific miRNAs with potential roles in transcript regulation.

## Discussion

The determination of a transcript’s protein-binding repertoire aids in elucidating regulatory mechanisms. However, so far, most studies for de-novo identification of transcript-bound proteins have focused on abundant non-coding or exogenously expressed RNAs and were only applied in cell culture^11–16^. Recently, a tandem purification approach for isolation of mRNA-protein complexes from yeast, *C. elegans*, and human cells has been proposed, but transcript enrichment has been reported to be limited^47^. Here, we present vIPR, a highly specific and sensitive method for de-novo identification of proteins interacting with an mRNA of interest in vivo in *C. elegans*.

Identification of proteins that bind to mRNAs in the context of an entire living animal is impeded by tissue complexity and a higher background of non-specific interactions. Generally, the success of an RNA pulldown experiment crucially depends on (1) the crosslinking efficiency (reduced in *C. elegans*), (2) RNA retrieval specificity and efficiency, (3) the signal-to-noise ratio, (4) sensitivity in mass spectrometry, and (5) stringent controls. We successfully identified interactomes of the *gld-1* and *lin-41* transcripts which are far less abundant than most previously analyzed RNAs and are exclusively or predominantly expressed in one tissue of *C. elegans*, the gonad. We sought to establish a method to find direct RNA binders. Both PAR-XL and cXL have been commonly used to generate covalent linkages between RNAs and directly bound proteins, while PFA-XL also crosslinks indirectly bound proteins. PAR-XL has successfully been applied to recover protein-RNA interactions for three different RBPs in *C. elegans*^18–20^, and enables mapping of RBP binding sites at nucleotide resolution. However, since PAR-XL depends on labeling efficiency and has a stronger nucleotide bias than cXL, and since crosslinking with cXL recovered known and anticipated binders of the *gld-1* transcript in our pilot experiment, we used cXL for all further experiments.

The signal-to-noise ratio is crucial for identification of transcript binders. Mass spectrometry has a limited dynamic range, implying that high amounts of background impede detection of lowly-abundant specific binders. Using vIPR, we achieved strong enrichment of the target RNA (Fig. 1b). Nevertheless, many specifically bound proteins were detected with few peptides only, while non-specific proteins were often detected at much higher peptide counts. This observation emphasizes that stringent controls are required to discriminate specific binders from background.

To control for non-specific binding, we performed vIPR with *gfp*-complementary probes in wild-type worms (no-target control). For ChIRP-MS and RAP-MS, several controls were tested that performed equally well in discriminating specific from non-specific binders^11,12^. In our experiments, choice of the control was crucial. Pulldown without prior crosslinking or pulldown after RNase treatment proved as inappropriate controls, presumably since these do not account for proteins crosslinked to background RNA. RAP-MS of the lncRNA Xist features transcript capture by ~140 90 nt long probes, with subsequent washes at elevated temperature under highly denaturing conditions^12^. The lower stringency of our washes, which is necessary to maintain transcript association of our 10–12 20 nt long probes, may contribute to the observed protein background in our no-target control. However, target RNA enrichment in vIPR experiments was comparable to the enrichment observed with RAP-MS^12^, suggesting that RNA background, and thus RNA-associated protein background, is similar with both approaches.

While performing pulldowns with a no-target control accounts for background inherent to the experimental setup, it does not enable discrimination of proteins binding specifically to the RNA of interest from proteins promiscuously binding to many RNAs without specific regulatory impact. Comparing vIPR of *gld-1::gfp* and *gfp::lin-41*, we indeed found that many proteins bind to both transcripts (Fig. 4a).

Both *gld-1* and *lin-41* are expressed non-uniformly along the germline^48,49^. Common binders may therefore represent regulators of both transcripts in specific stages of germ cell development. But also considering that all mRNAs go through the same initial processing events, the finding of common binders is not surprising. Interestingly, many of the identified candidates are described to at least transiently associate with P granules, dense assemblies of RNAs and proteins in the *C. elegans* germline. Most mRNAs transit through P granules when exported from the nucleus^50^, and it has been proposed that P granules provide an environment to facilitate coordinated RBP-mRNA interactions (reviewed in ref. ^51^). Consistently, *gld-1* mRNA was shown to partly reside in P granules and to depend on P granule localization for interaction with the protein FBF-2^52^.

The reproducibility of enriched binders of both transgenic and endogenous *gld-1* and *lin-41* pulldowns suggest that most enriched binders represent authentic in vivo interactors. However, we likely did not identify all transcript binders. PAR-XL and cXL were shown to recover different RBPs with different efficiencies^3^. Furthermore, some RBP classes, e.g., double-strand RBPs, are under-represented in UV-crosslink-based studies, and the sequence context of an RBP binding site can disfavor crosslinking^53^. The fact that we did not identify the known binder FBF in the *gld-1::gfp* triplicate pulldowns (Fig. 3a), but in all other *gld-1* pulldowns (Fig. 2d, Supplementary Fig. 4e), additionally indicates that further replicates and complementary experiments relying on other crosslinking methods than cXL may be necessary to comprehensively identify all interactors. Especially if interested in complex partners that do not directly bind the RNA of interest, it might be of advantage to use an approach based on PFA-XL. Notably, while many proteins are consistently enriched in the pulldowns of transgenic and endogenous *gld-1*, not all of them pass our defined enrichment and significance cut-offs. Thus, further binders might additionally be retrieved from the set of consistently identified proteins.

We independently validated the specific interaction of DAZ-1 with *gld-1* mRNA, as well as binding of TIAR-1, an RNA granule protein, to both *gld-1* and *lin-41*. Many RBPs regulate their own expression through feedback loops. Likewise, GLD-1 binds its own mRNA (this study;^19,54^). GLD-1 acts as a translational repressor and stabilizes a subset of targets^33,48,55^. We identified DAZ-1 as an additional regulator of the *gld-1* transcript. DAZ-1 is a germline-specific RBP important for oogenesis, and has been suggested to act as a translational activator^41,42,56^. Using a *gld-1* 3′ UTR reporter, we showed that protein expression was strongly downregulated upon *daz-1* knockdown (Fig. 4d, e). We speculate that DAZ-1 works together with GLD-1 to stabilize the *gld-1* transcript while concomitantly ensuring translation.

Applying vIPR, we not only identified protein binders, but additionally detected enriched miRNAs for the *lin-41, alg-1*, and *gld-1* transcripts. The fact that the recovered interactions were independently identified in miRNA:target chimera analyses^17,18^ suggests that they represent authentic in vivo interactions, and not post-lysis associations. Importantly, both the identified interactions of *lin-41* with *let-7* and *alg-1* with *miR-71* play crucial roles in development or aging^37,38,43^, demonstrating that vIPR enables discovery of miRNA:target interactions that are biologically relevant.

Downregulation of *lin-41* by *let-7* in somatic cells ensures proper developmental timing^37,38^. While somatic *lin-41* levels are low in adults, transcript levels are high in germ cells and it was suggested that *let-7* does not regulate *lin-41* in germ cells^49^. We found *let-7* to be enriched in *lin-41* vIPR experiments from young adult worms (Fig. 5a, Supplementary Fig. 5a). With our experimental setup, we cannot distinguish whether this enrichment represents functional interactions of *let-7* with the remaining somatic *lin-41* transcripts, or non-functional interactions within the germline. Investigating transcripts under control of tissue-specific promoters may help to resolve these scenarios.

The *gld-1* transcript is believed to be exclusively expressed in the germline, and we found *miR-84* to be specifically enriched in *gld-1* vIPR experiments. We validated the predicted binding site in the *gld-1* 3′ UTR by CRISPR editing of (1) the seed-complement and (2) bases pairing outside of the seed, both resulting in loss of *miR-84* enrichment (Fig. 5e, f). Further studies will be necessary to explore the consequences of the *miR-84:gld-1* interaction. Of note, the role and impact of miRNA regulation within *C. elegans* germ cells is still largely unclear^57^, and it seems that target repression is at least partly regulated by different mechanisms than in somatic cells^58^. Interestingly, miRNA targeting in the germline results in localization of mRNAs adjacent to perinuclear P granules, and this localization depends on the RNA helicase GLH-1^58^, a protein identified in pulldowns of both *gld-1* and *lin-41*. While we did not find the miRNA effector protein ALG-1, we found several of the proteins co-precipitating with miRNA complexes^58^ enriched in our pulldowns (3/3 germline-specific binders: GLH-1, CAR-1, GLD-1; 6/12 general binders: PAB-1, CGH-1, CEY-2, CEY-3, CEY-4, ZBP-1). Of note, ALG-1 has neither been identified in a recent study assessing the entire *C. elegans* mRNA interactome^4^, although ALG-1 CLIP has been performed successfully, and a large proportion of mRNAs are targeted by miRNAs^17,18,21^. This may be explained by a poor UV-crosslinkability of ALG-1. While a low crosslinking efficiency may suffice to detect crosslinked mRNAs in CLIP experiments, crosslinked RBPs may be missed in RNA pulldown experiments due to the lack of protein amplification methods and hence the lower sensitivity.

In conclusion, vIPR allows identification of both proteins and miRNAs binding to RNAs of interest in live *C. elegans*. We propose that mutation of an RBP binding site with subsequent vIPR may reveal changes in the composition of interacting proteins, thus providing insights into regulatory cascades. Furthermore, we anticipate application of vIPR to unravel differential regulation during development or in different cell types and of distinct transcript isoforms. We focused here on *gfp* transcripts for the ease of probe design and control. However, we demonstrated that endogenous transcripts can be retrieved similarly. vIPR should thus be readily applicable to any other similarly expressed *C. elegans* transcripts and we believe that it can be extended to discover interactions in any other animal or tissue amenable to UV crosslinking.

## Methods

### *C. elegans* maintenance

*C. elegans* strains (Supplementary Table 1) were cultivated using standard procedures. Worms were maintained at 24 °C on *Escherichia coli* OP50-seeded NGM plates^59^. The MosSCI injection strain EG6699 was kept at 16 °C for maintenance. The BS1080 *gld-1* transgenic strain^24^ was a gift from Tim Schedl. All other strains were obtained from the *Caenorhabditis* Genetics Center (CGC) or generated in the course of this study.

### RNA pulldown (vIPR)

Probe design: Probes (3′-biotin-TEG modified 20 nt long DNA antisense oligonucleotides; metabion) tiling the *gfp* CDS or entire endogenous transcripts were designed essentially as described previously^60^. Probes were mixed to obtain a final concentration of 100 μM (8.3 μM each for *gfp* probes; 10 μM each for endogenous probes). Sequences are listed in Supplementary Table 2.

Preparation of worm pellets: Arrested synchronized L1 larvae were generated by bleaching of gravid adult worms and o/n hatching of larvae in M9 (22 mM KH_2_PO_4_, 42 mM Na_2_HPO_4_, 86 mM NaCl, 1 mM MgSO_4_) without food source^59^. Experiments were typically performed with ~500,000 worms. Labeling of worms with the photoreactive nucleoside 4SU (Carbosynth, NT06186; final concentration 3 mM) was done in liquid culture (2000 worms per mL; 1 mL *of E. coli* OP50 with OD 2.3 per 1000 worms) at 24 °C, as described before^19^. Worms were harvested as young adults (typically after ~52–53 h), washed 3× in 0.1 M NaCl, transferred to non-seeded NGM plates and crosslinked in a custom UV Stratalinker 2400 crosslinker at a wavelength of 365 nm (energy: 3 J cm^−2^; ~16 min). For crosslinking methods not requiring labeling, worms were grown on OP50-seeded plates (45,000 worms per 15 mm plate) until reaching young adulthood (typically ~44–45 h). After three washes in 0.1 M NaCl, worms were either transferred to non-seeded NGM plates and crosslinked at a wavelength of 254 nm (Hoefer UV crosslinker UVC 500; energy: 1 J cm^−2^; ~2:45 min) or incubated for 30 min with 2% PFA in M9, with subsequent quenching by 0.1 M Tris–HCl, pH 7.6, and two more washes in 0.1 M NaCl. Worms were pelleted, buffer was removed, and crosslinked worm pellets were frozen in liquid nitrogen. Pellets were kept at −80 °C until processed further.

Worm lysis: After grinding worm pellets with mortar and pestle in liquid nitrogen, worm powder was resuspended in ~7× volume pulldown lysis buffer (50 mM Tris–Cl, pH 7.0, 10 mM EDTA, 1% SDS, 1 mM DTT, 1 mM PMSF, 1 μg mL^−1^ Pepstatin A, 1 tablet Complete EDTA-free Protease Inhibitor (Roche), 0.1 U μL^−1^ RiboLock RNase Inhibitor (Thermo Fisher Scientific)) and incubated for 30 min on ice. During incubation, lysates were passed 4× through a 20 gauge needle and 3× through a 25 gauge needle. For PFA-crosslinked samples, lysates were additionally sonicated (Sonicator HD2070, microtip MS72, Bandelin) with 7 W for 2 min (0.7 s on, 1.3 s off). All lysates were cleared by centrifugation (28,900 × *g*, 4 °C, 30 min), pellets were discarded, and supernatants filtered (0.2 μm Minisart syringe filters, Sartorius). Protein concentrations were determined using the Pierce BCA Protein Assay Kit (Thermo Fisher Scientific), and adjusted with lysis buffer, so that all samples processed simultaneously had the same final concentration (typically 2–3.5 μg μL^−1^). Lysates were diluted by adding 2× volume of pulldown hybridization buffer (750 mM NaCl, 1% SDS, 50 mM Tris–Cl, pH 7.0, 1 mM EDTA, 15% formamide), and input samples were taken for RNA and protein analysis. The pulldown procedure was adapted from the protocol of Chu and colleagues^60^, with modifications.

Preparation of beads and preclearing: For pulldown with subsequent mass spectrometry, lysates were precleared prior to pulldown. MyOne Streptavidin C1 magnetic beads (Thermo Fisher Scientific; 100 μL per 1 mL lysate with protein concentration 2 μg μL^−1^) were washed 3× in 1× original volume lysis buffer, resuspended in 0.5× volume lysis buffer, and added to the lysate. Preclearing was done at 37 °C for 1–2 h under constant rotation. To ensure complete removal of beads from lysates, tubes were placed on a magnet (DynaMag-15, Thermo Fisher Scientific) and lysates were transferred to new tubes twice.

Probe hybridization and capture: Probes (50 pmol per 1 mL lysate with protein concentration 2 μg μL^−1^) were added to lysates and samples were incubated at 37 °C for 2 h or o/n. For probe capture, MyOne C1 beads, prepared as above, were added (100 μL per 50 pmol probes) and samples were incubated for an additional hour at 37 °C. Beads were separated from lysate and supernatant samples were taken. Beads were washed 5× with ~13 mL wash buffer (2× SSC, 0.5% SDS). Finally, beads were resuspended in 1 mL wash buffer and transferred to Protein LoBind tubes (Thermo Fisher Scientific). A 50–100 μL aliquot was used for RNA isolation (DNA LoBind tubes, Thermo Fisher Scientific). Wash buffer was removed via magnet (DynaMag-2, Thermo Fisher Scientific), and beads resuspended in Benzonase elution buffer (10 mM Tris–Cl, pH 7.5, 1 mM MgCl_2_, 1 mM DTT, 0.625 U μL^−1^ Benzonase (Millipore, 71205-3)) for protein elution and Proteinase K buffer (100 mM NaCl, 10 mM Tris–Cl, pH 7.0, 1 mM EDTA, 0.5% SDS, 1 mg mL^−1^ Proteinase K (Roche)) for RNA isolation, respectively.

RNA isolation: Beads, input and supernatant samples were resuspended in 100 μL Proteinase K buffer, and incubated at 50 °C for 45 min, shaking at 1300 rpm. Proteinase K was inactivated by boiling at 95 °C for 10 min. Samples were chilled on ice, and 1 μL GlycoBlue (ThermoFisher Scientific) was added, followed by Trizol RNA isolation (Thermo Fisher Scientific).

Protein elution: Input and supernatant samples (10 μL) were resuspended in 100 μL and beads in 200 μL Benzonase elution buffer. Crosslinked proteins were eluted by incubation at 37 °C for 3 h, shaking at 1300 rpm. Beads were separated from elution via magnet, and elution was transferred to new Protein LoBind tubes. Samples were snap-frozen in liquid nitrogen and kept at −80 °C until processed further.

### Sample preparation for mass spectrometry

For input and supernatant samples, 6–13 μg of protein were used for analysis by mass spectrometry. For pulldown elution samples, we roughly estimated the protein amount to be ~250 ng, based on comparison of peptide intensities of Trypsin and Benzonase in input and elution samples with corresponding total peptide intensities. Proteins were precipitated with ethanol and resuspended in 50 μL of 8 M urea and 0.1 M Tris–HCl, pH 8. Proteins were reduced with 10 mM DTT at room temperature for 30 min and alkylated with 50 mM iodoacetamide at room temperature for 30 min in the dark. Proteins were first digested by LysC (Wako) at a LysC-to-protein ratio of 100:1 at room temperature for 3 h. Then, the sample solution was diluted to a final concentration of 2 M urea with 50 mM ammonium bicarbonate. Trypsin (Promega) digestion was performed at a trypsin-to-protein ratio of 100:1 under constant agitation at room temperature for 16 h. Digestion was stopped and pH adjusted to <3.0 with TFA. Peptides were desalted with SCX (strong cation chromatography) and C18 Stage Tips^61^ prior to nanoLC–MS/MS analysis.

### NanoLC–MS/MS analysis

Reversed-phase liquid chromatography (rpHPLC) was performed employing an EASY nLC II (Thermo Fisher Scientific) using self-made fritless C18 microcolumns (^62^; 75 μm ID packed with ReproSil-Pur C18-AQ 3-μm or 1.9-μm resin, Dr. Maisch) connected on-line to the electrospray ion source (Proxeon) of a Q Exactive plus or a Q Exactive HF-X mass spectrometer (Thermo Fisher Scientific). Peptide samples were eluted at a flow rate of 250 nL min^−1^ with a 5–48% acetonitrile gradient in 0.1% formic acid over 2 h. Settings for MS analysis for Q Exactive Plus were as follows: one full scan (resolution 70,000; *m*/*z* 300–1700) followed by top 10 MS/MS scans using higher-energy collisional dissociation (HCD) (min. signal required, 21,000; isolation width, 2 *m*/*z*; normalized collision energy, 26). Settings for MS analysis for Q Exactive HF-X were: one full scan (resolution 60,000; *m*/*z* 350–1800) followed by top 20 MS/MS scans using HCD (min. signal required, 21,000; isolation width, 1.3 *m*/*z*; normalized collision energy, 26). Ions with an unassigned charge state and singly charged ions were rejected. Former target ions selected for MS/MS were dynamically excluded for 30 s.

### CLIP-qPCR

CLIP-qPCR was performed to validate candidate interactors identified with vIPR. For reproducible and efficient immunoprecipitation, we used transgenic *C. elegans* strains expressing GFP-tagged proteins, together with α-GFP Trap_A beads (chromotek, gta-20). Worms (typically ~25,000) were synchronized and grown until reaching young adulthood. After harvesting, worms were washed 3× with 0.1 M NaCl. Worms were transferred to non-seeded NGM plates, crosslinked at 254 nm (energy: 1 J cm^−2^), pelleted, and snap-frozen in liquid nitrogen. Pellets were kept at −80 °C until processed further. For the CLIP experiment, worm pellets were resuspended in 1 mL NP-40 lysis buffer (50 mM HEPES, pH 7.4, 150 mM KCl, 0.5% (v/v) NP-40, 2 mM EDTA, 0.5 mM DTT, Complete mini EDTA-free Protease Inhibitor (Roche), 1 µg mL^−1^ Pepstatin, 1 mM PMSF, 0.1 U µL^−1^ RiboLock RNase Inhibitor (Thermo Fisher Scientific)) and homogenized with SiLibeads (Sigmund Lindner) in a tissue lyzer (precellys 24 homogenizer, Bertin Technologies; (6000 rpm, 2 × 10 s). Lysates were incubated on ice for 20–30 min, passed 10× through a 20 gauge needle, cleared by centrifugation (~16,100 × *g*, 4 °C, 20 min), and filtered (0.2 µm Minisart syringe filters, Sartorius). After taking input samples, lysates were added to previously equilibrated α-GFP Trap_A beads (25 µL bead slurry per sample) for immunoprecipitation (90 min at 4 °C). After collection by centrifugation, beads were washed 4× with NP-40 lysis buffer and 2× with high-salt IP wash buffer (50 mM HEPES, pH 7.4, 300 mM KCl, 0.05% (v/v) NP-40, 2 mM EDTA, 0.5 mM DTT, Complete mini EDTA-free Protease Inhibitor, 1 µg mL^−1^ Pepstatin, 1 mM PMSF). Beads and input samples were resuspended in 100 μL Proteinase K buffer (100 mM NaCl, 10 mM Tris–Cl, pH 7.0, 1 mM EDTA, 0.5% SDS, 1 mg mL^−1^ Proteinase K (Roche)), and incubated at 50 °C for 45 min, shaking at 1300 rpm. Proteinase K was inactivated by boiling at 95 °C for 10 min. Samples were chilled on ice, and 1 μL GlycoBlue (ThermoFisher Scientific) was added, followed by Trizol RNA isolation (Thermo Fisher Scientific).

### RT-qPCR

For quantification of RNA levels by qPCR, 1 µg of HEK RNA was added to samples prior to RNA isolation. Extracted RNA was RQ1 DNase-treated (Promega), and cDNA was generated with random hexamer primers, dNTPs and Maxima H Minus Reverse Transcriptase (Thermo Fisher Scientific). qPCRs were performed with Maxima SYBR Green/ROX qPCR Master Mix (Thermo Fisher Scientific) and 1/5 dilutions of cDNA as template. Oligonucleotides used for qPCRs are listed in Supplementary Table 3.

### Total RNA library preparation and sequencing

To extract total RNA from worms, worms were washed in 0.1 M NaCl, resuspended in 1 mL Trizol reagent (Thermo Fisher Scientific), and homogenized with SiLibeads (Sigmund Lindner) in a tissue lyzer (precellys 24 homogenizer, Bertin Technologies; 6000 rpm, 2 × 10 s), followed by RNA extraction according to the Trizol protocol. Pulldown elution samples for RNA sequencing were supplemented with 1 μg HEK RNA before RNA isolation to enhance the efficiency and reproducibility of RNA precipitation. Additionally, samples from PAR-XL and PFA-XL pulldowns contained ERCC RNA Spike-In Mix 1 (Thermo Fisher Scientific, 4456740). After Trizol RNA isolation, ribosomal RNAs were depleted by one of the methods described below.

rRNA depletion by RNase H treatment: Ribosomal RNA depletion with RNase H was essentially performed as described by Adiconis et al.^63^. Briefly, 500 ng RNA were mixed with 500 ng ribosomal RNA-complementary DNA oligonucleotides (for input samples: *C. elegans* ribosomal RNAs; for elutions samples: human ribosomal RNAs) in hybridization buffer (0.2 M NaCl, 0.1 M Tris–HCl, pH 7.6), heated at 95 °C for 2 min and cooled to 45 °C at a rate of −0.1 °C s^−1^ for specific annealing of oligonucleotides to ribosomal RNA. Samples were supplied with an equal volume of RNase H buffer (1 μL Hybridase RNase H (Lucigen) per 500 ng RNA, 0.2 M NaCl, 0.1 M Tris–HCl, pH 7.6, 40 mM MgCl_2_), mixed and incubated for additional 30 min at 45 °C for ribosomal RNA depletion. RNA was extracted with Agencourt RNAClean XP beads (Beckman Coulter) and TURBO DNase-treated (Thermo Fisher Scientific) for 30 min at 37 °C to remove DNA oligonucleotides. Reaction was stopped by addition of EDTA to a final concentration of 15 mM and incubation at 75 °C for 10 min. RNA was purified a second time by RNAClean XP beads and resuspended in 19.5 μL of Elute, Prime, Fragment Mix from the TruSeq RNA library prep kit v2 (Illumina).

rRNA depletion with RiboMinus kit: Elution RNA of PAR-XL and PFA-XL pulldowns was TURBO DNase-treated for 30 min at 37 °C. Reaction was stopped by addition of EDTA to a final concentration of 15 mM and incubation at 75 °C for 10 min. RNA was purified by RNAClean XP beads, and ribosomal RNA depletion performed with the RiboMinus eukaryote probe mix v2 (Thermo Fisher Scientific), according to the manufacturer’s protocol. After depletion, RNA was ethanol-precipitated (o/n), and resuspended in 19.5 μL Elute, Prime, Fragment Mix from the TruSeq RNA library prep kit v2.

Library preparation and sequencing: All samples were subjected to fragmentation, reverse transcription and adapter ligation, as described in the TruSeq RNA sample preparation v2 guide. By means of a pilot qPCR with 1/10th of generated libraries and library- and adapter-specific primers, the optimal Ct for library amplification was determined. Library quality, quantity and average fragment sizes were assessed by Bioanalyzer 2100 (DNA 1000 kit, Agilent) and Qubit (Thermo Fisher Scientific). Samples were sequenced in 1 × 76 run mode on a NextSeq 500 system (Illumina) or 1 × 51 run mode on a HiSeq 4000 system (Illumina).

### Small RNA library preparation and sequencing

RNA from target and no-target pulldowns was extracted with Trizol reagent, after addition of 1 µL GlycoBlue (Thermo Fisher Scientific). RNA was RQ1 DNase-treated (Promega), and purified with Roti-Aqua-PCI (Carl Roth), followed by ethanol precipitation. RNA was resuspended in 8.5 µL H_2_O, and 7 µL were used for library preparation with the SMARTer smRNA-Seq Kit for Illumina (Clontech). After PCR purification, the library concentration was measured with Qubit (Thermo Fisher Scientific) and size distribution was checked by Bioanalyzer (DNA HS chip, Agilent). The libraries were size-separated via BluePippin (sage science; 3% gel, marker Q2), according to the SMARTer smRNA-Seq Kit protocol. Size-selected libraries were again analyzed by Bioanalyzer (HS DNA chip). Equimolar amounts of libraries were pooled and loaded onto a NextSeq 500 (Illumina) in 1 × 76 run mode or a HiSeq 4000 (Illumina) in 1 × 51 run mode.

### Reporter cloning and generation of transgenic lines

The reporter construct was generated by conventional restriction-ligation cloning, as described before^64^. The final construct contained the *gld-1* promoter and 5′ UTR, the GFP::H2B CDS, and the *gld-1* 3′ UTR within the backbone of the vector pCFJ151. The transgenic strains were generated by the MosSCI technique^65,66^, resulting in single-copy insertion of the transgene at a defined locus on chromosome II of the *C. elegans* genome. Oligonucleotides used for construct cloning are listed in Supplementary Table 4.

### RNA interference

Worms were synchronized by bleaching, and seeded on RNAi feeding plates^59^. Mock (L4440 empty vector) and *daz-1* (sequence name in library: F56D1.5) RNAi clones were obtained from the Ahringer library. Per well in a 6-well plate, 400 L1 animals were seeded. For analysis of RNA levels, young adult worms were harvested after growth at 24 °C for 45 h, washed in 0.1 M NaCl, and resuspended in 500 µL Trizol reagent (Thermo Fisher Scientific). After homogenization with SiLibeads (Sigmund Lindner) in a tissue lyzer (precellys 24 homogenizer, Bertin Technologies; 6000 rpm, 2 × 10 s), insoluble material was pelleted by centrifugation for 5 min at 16,100 × *g*. From the supernatant, 350 µL were used for RNA isolation by means of the Direct-zol kit (Zymo Research), which includes a DNase digestion step. Of the purified RNA, 100 ng were used as input for amplification-free RNA quantification by the Nanostring nCounter gene expression assay using a 72-multiplex custom Nanostring Gene Expression code set on a nCounter SPRINT profiler (Nanostring Technologies) following the manufacturer’s instructions. Raw counts were normalized to the internal positive controls and to two reference genes (*tbb-1*, *tbb-2*), using the nSolver 4.0 software (Supplementary Data 5). Imaging of reporter GFP expression upon RNAi was done in a time window between 45 and 50 h after seeding, and is described below.

### Imaging and quantification of fluorescence

Young adult worms were mounted by picking clean worms into a drop of 1 mM levamisole in M9 (22 mM KH_2_PO_4_, 42 mM Na_2_HPO_4_, 86 mM NaCl, 1 mM MgSO_4_) on a 2% agarose pad. Pictures of GFP expression in gonads were taken on an inverted fluorescence microscope (BZ-X710, Keyence) with a 40× objective (Plan Apo λ 40×/0.95; gain disabled; 2 × 2 binning). All gonads were monitored using the same exposure time (1/7.5 s). Pictures were processed and analyzed using ImageJ in an identical manner. Germline GFP fluorescence was quantified by measuring pixel intensity profiles with ImageJ along a segmented line (thickness: 35) from the distal tip to the bend of each gonad arm as described previously^54,64^. Background mean gray values were subtracted, and values were binned into 30 bins. Averages and standard error of the mean (SEM) were calculated for all gonads analyzed per condition and independent reporter line. Quantification was restricted to distal gonads as absence of DAZ-1 leads to meiosis arrest of oogenic germ cells at the pachytene stage^56^.

### Genome editing

Genome editing for destruction and conversion of the *miR-84* binding site in the *gld-1* 3′ UTR was essentially performed as described by Paix and colleagues^67^. The tracrRNA (42.5 µM; Alt-R® CRISPR-Cas9 tracrRNA, IDT) was pre-annealed with the target crRNA (30 µM; Alt-R® CRISPR-Cas9 crRNA, IDT) and the *dpy-10* crRNA (12 µM; Ce.Cas9.DPY-10.1.AQ, Alt-R^®^ CRISPR-Cas9 crRNA, IDT) by incubation at 95 °C for 5 min and cooling to 25 °C at a rate of −0.1 °C s^−1^. Recombinant Cas9 protein (9 µg) was mixed with the pre-annealed RNA, the target repair ssODN (760 nM; Ultramer DNA Oligo, IDT) and the *dpy-10* ssODN (440 nM; Ultramer DNA Oligo, IDT) in a solution containing 150 mM KCl and 7.5 mM HEPES, pH 7.4 (final volume 10 µL). The mix was injected into gonads of adult wild-type N2 worms. Sequences of oligonucleotides used for CRISPR are listed in Supplementary Table 5.

### RNA mapping and quantification

After de-multiplexing (bcl2fastq Conversion Software v2.17.1.14, Illumina), sequencing reads were pseudo-aligned with kallisto (^68^; version: 0.43.1; parameters: reads = single end, bootstraps = 100) to the *C. elegans* transcriptome (Caenorhabditis_elegans.WBcel235, Ensembl release v81), and if applicable to the *gld-1::gfp* or *gfp::lin-41* transgene sequence (determined by Sanger sequencing), and the ERCC RNA Spike-Ins. For pulldown elution samples that contained HEK RNA, the human transcriptome (GENCODE Release 24) was added to the index. Average fragment length and corresponding standard deviation were set according to the respective library Bioanalyzer profiles. Ribosomal RNAs (and overlapping pseudogenes) and, if applicable, human RNAs and ERCC Spike-Ins, were removed from output, and TPMs were re-normalized to the remaining transcripts. TPMs were summed up per gene, and plotted with the R software package.

### Identification and enrichment analysis of miRNAs

After de-multiplexing (bcl2fastq Conversion Software v2.18.0.12, Illumina), adaptors and poly(A)-tails were removed using cutadapt (version 1.14) and the following parameters --format = fastq --adapter = AAAAAA --error-rate = 0.2 --times = 3 --overlap = 3 --cut = 3 --quality-cutoff = 20 --quality-base = 33 --minimum-length = 17 --max-n = 0 --discard-untrimmed. The adaptor- and poly(A)-trimmed libraries were subsequently mapped with bowtie2 (version 2.3.3.1) using the parameters --very-fast-local --phred33 --local to the *E. coli* genome (NC_000913.3, K-12, MG1655) for removal of *E. coli* RNA contamination. miRNAs were then identified by miRDeep2 (^69^; version 2.0.0.7) with the miRBase21 reference. For miRNA enrichment analysis, raw read counts were plotted with the R software package and enrichment was determined by comparing regression-normalized miRNA counts.

### Protein identification and quantification

Raw data were analyzed and processed using MaxQuant (^70^; v1.5.1.2, 1.5.7.4, or 1.6.0.16) with standard settings, unless stated otherwise in the following. Search parameters included two missed cleavage sites, fixed cysteine carbamidomethyl modification, and variable modifications including methionine oxidation, N-terminal protein acetylation, and asparagine/glutamine deamidation. The peptide mass tolerance was 6 ppm for MS scans and 20 ppm for MS/MS scans. The match between runs option was enabled. Database search was performed using Andromeda^71^ against the UniProt/Swiss-Prot worm database (October 2014; April 2017) with common contaminants. The false discovery rate (FDR) was set to 1% at both peptide and protein level. Protein quantification was done based on razor and unique peptides. The label-free algorithm^34^ based on peptide extracted ion chromatograms (XICs) was used. The minimum LFQ ratio was set to 1 (vIPR triplicates of transgenic transcripts) or 2 (vIPR of endogenous transcripts). For analysis of identified proteins, known contaminants, proteins only identified by site, and reverse mappings were filtered out from MaxQuant output. For pulldown samples, we additionally removed proteins with gene ontology (GO) terms related to “biotin” (PYC-1, MCCC-1, POD-2, PCCA-1, T28F3.5/C1P655, BPL-1), which are expected to enrich unspecifically due to direct binding to the streptavidin-coated beads. For the initial performance test of crosslinking methods, we compared raw peptide intensities. For more accurate quantification of the cXL vIPR triplicate experiments, we compared LFQ intensities. Here, imputation of missing intensities was done with the Perseus software package (^72^; version 1.5.6.0), after log_2_-transformation of LFQ values (normal distribution, width: 0.3; shift: 1.8). To determine significance of proteins identified in triplicate, we calculated *p*-values with a moderated *t*-test, implemented in the Bioconductor LIMMA package^73^, and corrected for multiple comparisons by the Benjamini-Hochberg procedure^74^.

### GO term analysis

Majority protein IDs from MaxQuant output were mapped to Wormbase IDs by Uniprot ID mapping (http://www.uniprot.org/uploadlists/) and curated manually. Wormbase IDs were then used to retrieve gene ontology (GO) terms. Test for over-representation (PANTHER Overrepresentation Test, Released 20171205) was performed by the online tool at http://pantherdb.org/ (^75^; results in Supplementary Data 4). Over-representation was analyzed comparing consistently enriched genes with all genes reproducibly identified in all three independent *gld-1::gfp* pulldowns. Of 271 genes, 269 were retrieved and had annotated GO terms.

### Reporting summary

Further information on research design is available in the Nature Research Reporting Summary linked to this Article.

## Supplementary Information

Supplementary Information

Peer Review

Reporting Summary

Description of Additional Supplementary Files

Supplementary Data 1

Supplementary Data 2

Supplementary Data 3

Supplementary Data 4

Supplementary Data 5

Supplementary Data 6

Source Data

## Data Availability

A Reporting Summary for this Article is available as a Supplementary Information file. Raw RNA sequencing data has been deposited under the GEO accession code GSE130733. The mass spectrometry proteomics data have been deposited to the ProteomeXchange Consortium via the PRIDE^76^ partner repository with the dataset identifier PXD013720. All processed and analyzed RNA sequencing and mass spectrometry data have been provided in the Supplementary Data. The source data underlying Figs. 1b, 4b, c, e and Supplementary Figs. 1c, h, i and 4c, h are provided as a Source Data file. All data is available from the corresponding authors upon reasonable request.
