# Supplementary material for: Identification of proteins and miRNAs that specifically bind an mRNA in vivo
Source: Nat Commun. 2019 Sep 16;10:4205. doi: 10.1038/s41467-019-12050-7 (PMC6746756; doi:10.1038/s41467-019-12050-7)
Supplement: Supplementary file 1 — Supplementary Information [file 41467_2019_12050_MOESM1_ESM.pdf]

Supplementary Information

Identification of proteins and miRNAs that specifically bind an mRNA *in vivo*

Theil *et al.*

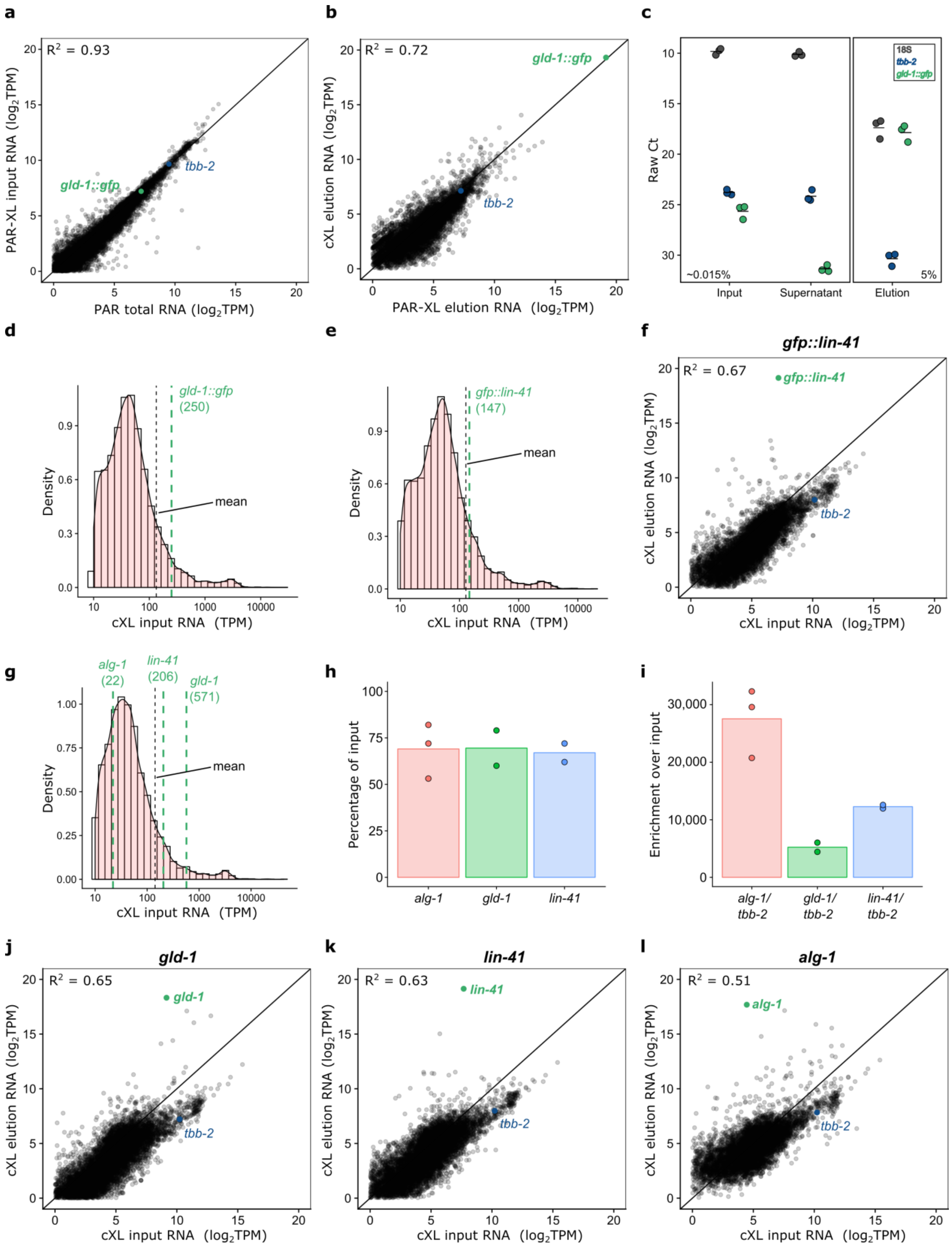

Supplementary Figure 1. Pulldown lysis conditions do not alter relative transcript counts and allow specific enrichment of both transgenic and endogenous RNAs. a) RNA from 4-thiouridine (4SU) labeled worms was either isolated directly (PAR total RNA) or after crosslinking and lysis within the pulldown procedure (PAR-XL input RNA). Plotted are gene transcripts detected with a TPM count >1 in both RNA sequencing libraries. Solid line represents diagonal. See also **Supplementary Data 1**. **b)**

RNA sequencing from PAR-XL and cXL vIPR shows reproducible RNA enrichment. **c)** Comparison of raw Ct values of the RNA of interest (*gld-1::gfp*) and control RNAs (*tbb-2* mRNA, 18S ribosomal RNA) in pulldown input, supernatant, and elution samples, measured by RT-qPCR. Samples from n = 3 independent pulldowns (one replicate per crosslinking method – PAR-XL, cXL, PFA-XL). **d,e)** Expression distribution of protein-coding transcripts with TPM counts >10 in pulldown input RNA from the *gld-1::gfp* strain (**d**) and the *gfp::lin-41* strain (**e**). **f)** RNA sequencing of input and pulldown elution samples confirms, transcriptome-wide, the specific enrichment of the *gfp::lin-41* transcript. Plotting analogous to **a**. **g)** Expression distribution of protein-coding transcripts with TPM counts >10 in pulldown input RNA from wild-type worms. **h)** vIPR leads to efficient capture of all tested endogenous transcripts. Bars represent means, data points represent n = 3 (*alg-1*), n = 2 (*gld-1*), and n = 2 (*lin-41*) independent pulldown experiments. **i)** Enrichment of endogenous target transcripts over the control *tbb-2*, comparing elution with input RNA. RNA levels were measured by RT-qPCR from n = 3 (*alg-1*), n = 2 (*gld-1*), n = 2 (*lin-41*) independent pulldown experiments, bars represent means. **j-l)** RNA sequencing of pulldown input and elution samples confirms, transcriptome-wide, specific enrichment of the tested endogenous target transcripts *gld-1* (**j**), *lin-41* (**k**), and *alg-1* (**l**). Plotting analogous to **a**. Source data are provided as a Source Data file.

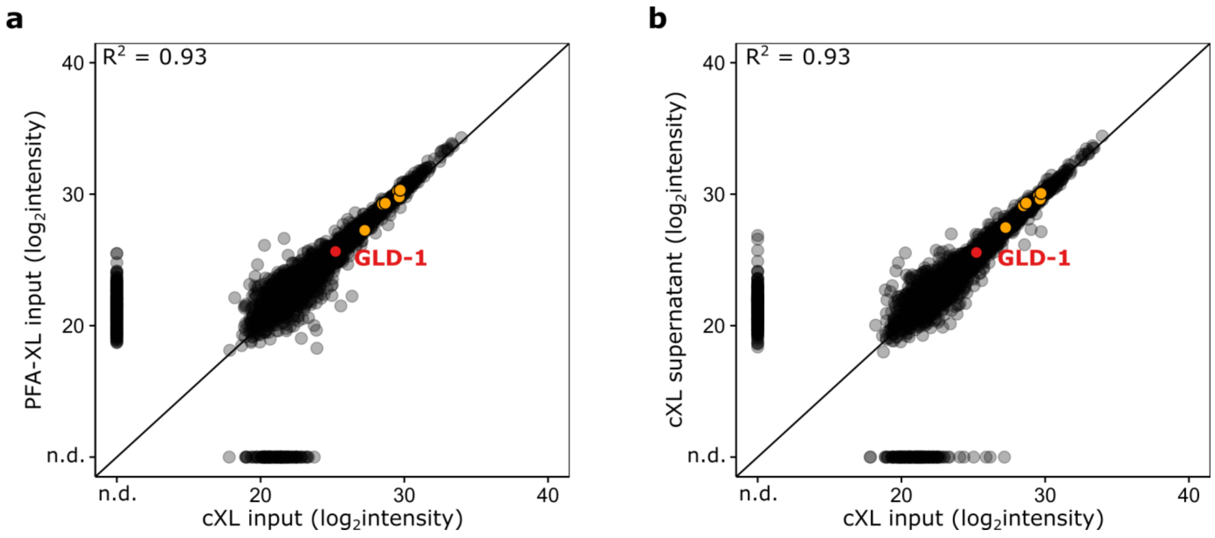

Supplementary Figure 2. Intensities of detected proteins are independent of crosslinking methods and reproduced after pulldown. a) Peptide raw intensities for proteins identified in input samples from vIPR with cXL versus vIPR with PFA-XL. **b)** Peptide raw intensities from input versus supernatant of vIPR with cXL. To calculate R^2, only proteins detected in both samples were considered. Red dot indicates the known binder GLD-1, yellow dots indicate proteins known to co-precipitate with GLD-1 protein (compare **Fig. 2e**), n.d.: not detected. See also **Supplementary Data 2** for peptide counts and intensities.

a **b**

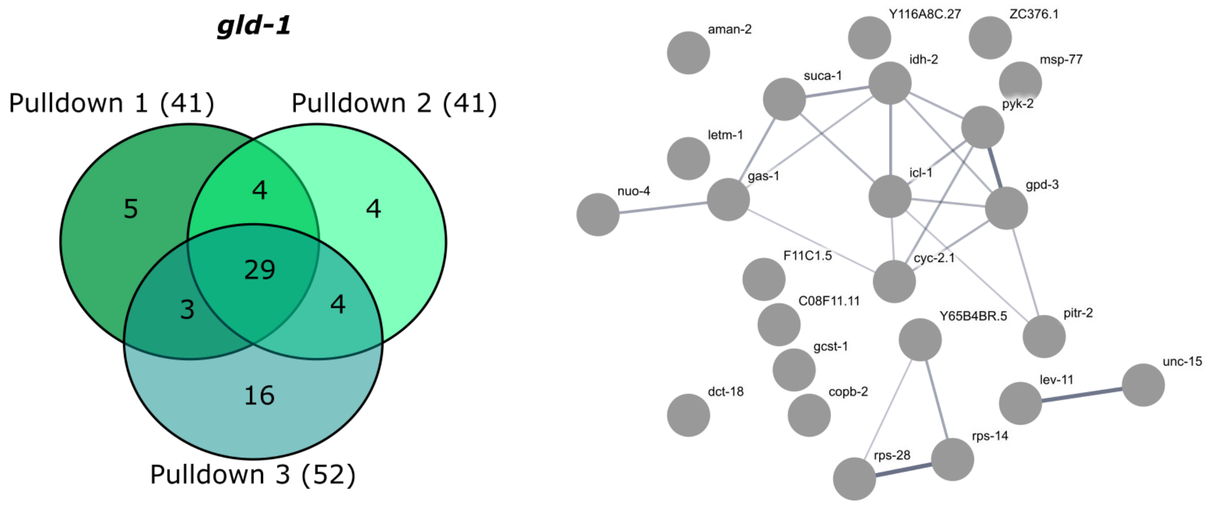

Supplementary Figure 3. vIPR of *gld-1::gfp* leads to reproducible protein enrichment. a) Shown are proteins that were reproducibly detected in three independent *gld-1::gfp* pulldowns and were found >4-fold enriched versus the respective negative control in at least one pulldown. Many proteins (n = 29) were consistently enriched in all replicates. **b)** Representative example of a STRING network for background binders in *gld-1::gfp* vIPR. The sample of 24 proteins was randomly selected from the set of proteins reproducibly detected in *gld-1::gfp* vIPR, but not enriched versus no-target control. Compare to **Fig. 3b**. The number of edges is much lower than for the enriched proteins (*p*-value < 7.65×10^{-7}).

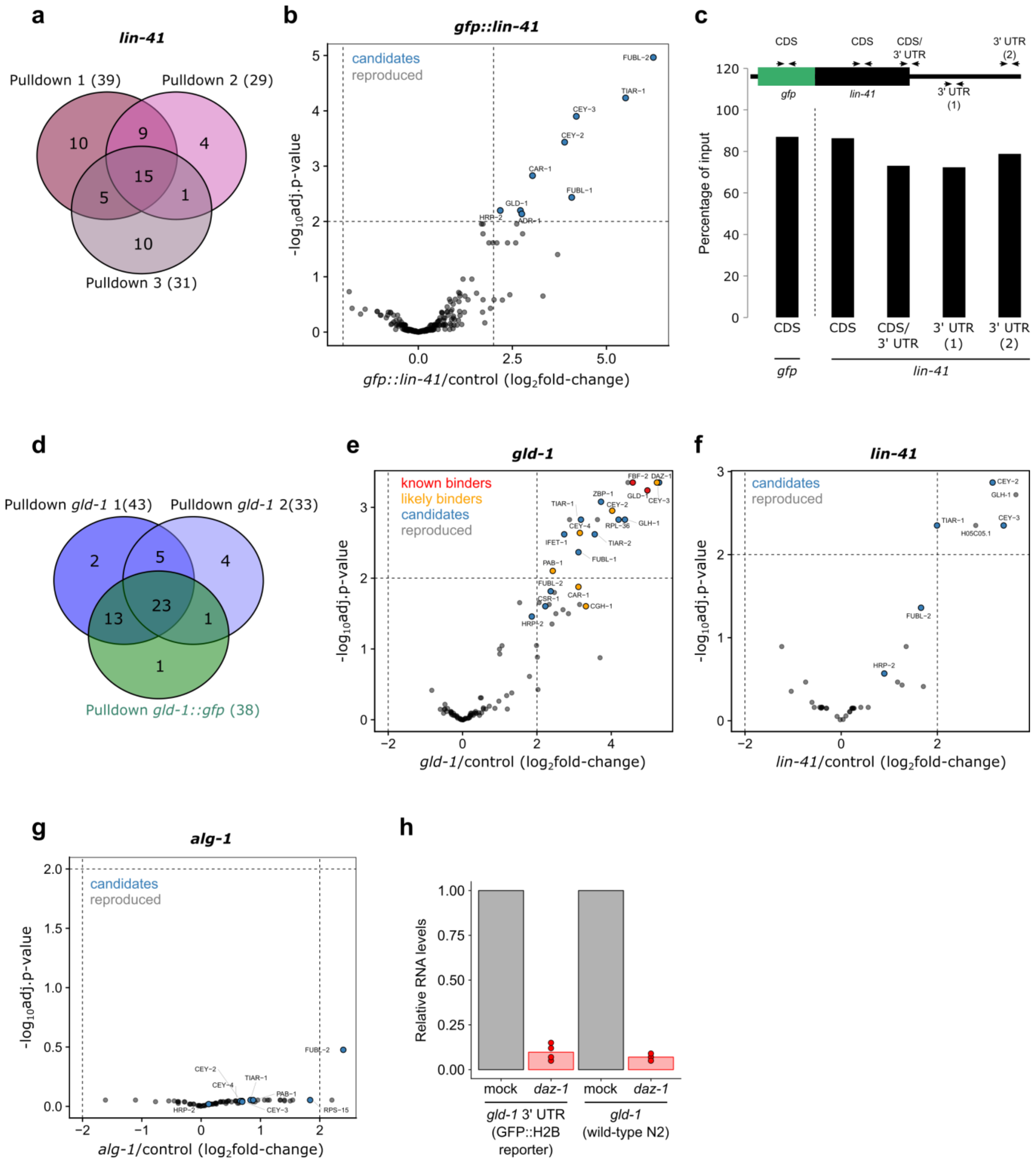

Supplementary Figure 4. vIPR leads to reproducible protein enrichment for both transgenic and endogenous transcripts. a) Shown are proteins that were reproducibly detected in three independent *gfp::lin-41* pulldowns and were found >4-fold enriched versus the respective negative control in at least one pulldown. Many proteins (n = 15) were consistently enriched in all replicates. **b)** vIPR of *gfp::lin-41* leads to reproducible enrichment of proteins versus no-target control. Proteins reproducibly detected: n = 278, proteins reproducibly enriched: n = 9. Mean fold-changes and *p*-values (moderated *t*-test; BH-corrected; **Supplementary Data 3**) from three independent pulldown experiments are plotted. Significance cut-off: adjusted *p*-value < 0.01, enrichment cut-off: >4-fold. **c)** The pulldown procedure efficiently captures all regions of the *gfp::lin-41* transcript. Comparison of retrieval of the *gfp* CDS and indicated transcript regions in elution sample, measured by RT-qPCR. Schematic on top shows transcript regions with respective primer pairs for amplification. **d)** Shown

are proteins that were reproducibly detected in one *gld-1::gfp* and two independent *gld-1* pulldowns and were found >4-fold enriched versus the respective negative control in at least one pulldown. Many proteins (n = 23) were consistently enriched in all replicates. **e,f)** vIPR of *gld-1* (**e**) and *lin-41* (**f**) leads to reproducible enrichment of proteins versus control. Mean fold-changes and *p*-values (moderated *t*-test; BH-corrected; **Supplementary Data 3**) from three independent pulldown experiments (one for the *gfp*-tagged and two for the untagged, endogenous transcripts) are plotted. Significance cut-off: adjusted *p*-value < 0.01, enrichment cut-off: >4-fold. **g)** vIPR of *alg-1* does not yield reproducibly detected proteins that pass the significance and enrichment cut-offs (three independent pulldowns of the endogenous *alg-1* transcript). **h)** RNA levels of *daz-1* in mock and *daz-1* RNAi treated young adult worms, measured by the Nanostring nCounter technology, and normalized to the controls *tbb-1* and *tbb-2*. Data from n = 4 (*gld-1* reporter) and n = 3 (wild-type N2) independent biological replicates, bars represent means. See also raw data in **Supplementary Data 5**. Source data are provided as a Source Data file.

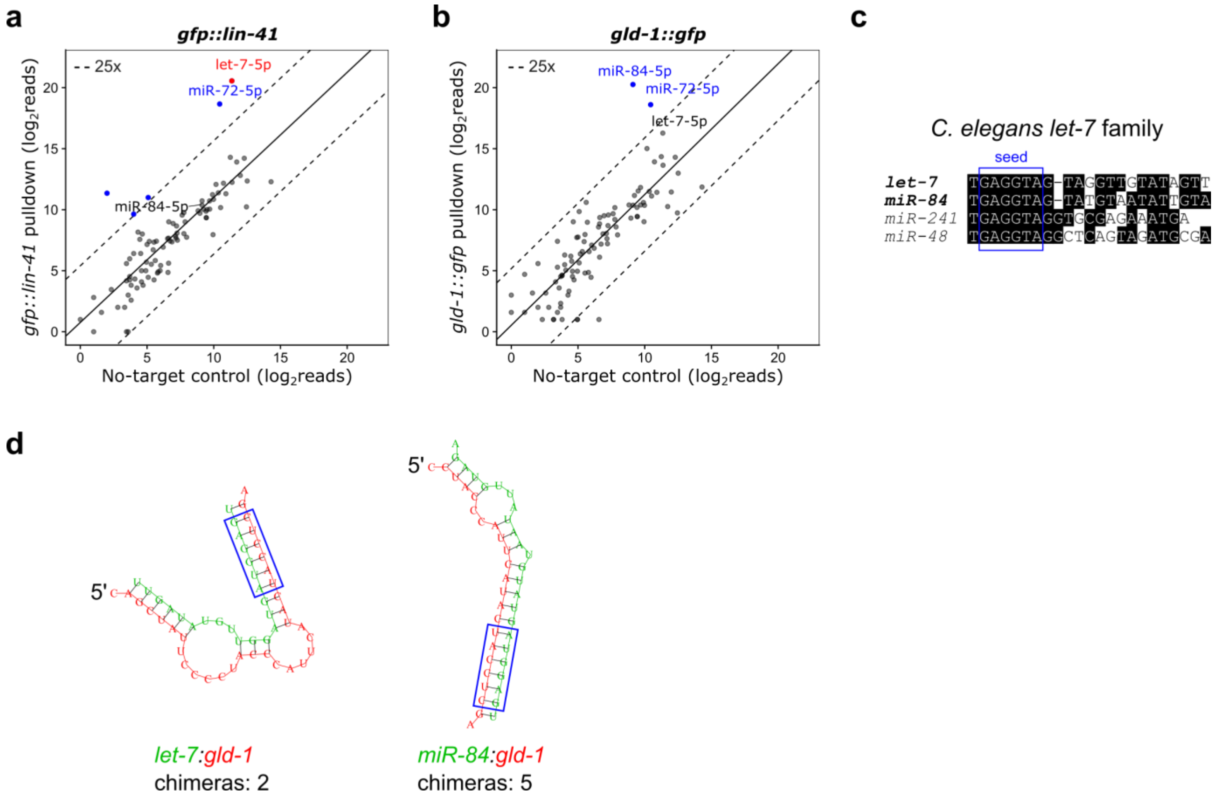

Supplementary Figure 5. Identification of transcript-specific miRNAs of *gfp*-tagged mRNAs. a,b) vIPR identifies miRNAs that specifically enrich versus no-target control (blue dots) for both the *gfp::lin-41* (**a**) and the *gld-1::gfp* (**b**) transcripts. Known regulators are colored in red. Plotted are miRNAs found in both target and no-target pulldown. Solid line: linear regression of $\log_2$-transformed reads. For *gld-1::gfp*, data from one of two independent pulldown experiments is shown. **c)** Sequence alignment of the *C. elegans let-7* miRNA family. *let-7* and *miR-84* feature a high sequence similarity. **d)** RNAhybrid [1] predictions for *let-7* and *miR-84* binding to the *gld-1* 3' UTR. Below, the number of chimeric reads from an ALG-1 CLIP study [2] supporting the predicted pairing region are given. Chimeras are ligation products of miRNAs and mRNAs found in Argonaute CLIP experiments, and are indicative of direct miRNA-mRNA interactions. Green marks miRNAs, red marks mRNAs, the target-paired miRNA seed sequence is highlighted with blue boxes. See also **Supplementary Data 6** for miRNA reads.

Supplementary Table 1. ***C. elegans*** **strains used in this study**

**Strain**	**Genotype**	**Used for**
BS1080	ozIs5 [gld-1p::gld-1::GFP::FLAG ORF::gld-1 3'utr + unc-119(+)] I	vIPR, CLIP-qPCR
DG3913	lin-41(tn1541[GFP::tev::s::lin-41]) I	vIPR, CLIP-qPCR
JH2119	unc-119(ed3) III; axIs1533[pie-1p::GFP::daz-1 ORF::daz-1 3'utr + unc-119(+)]	CLIP-qPCR
DG3922	tiar-1(tn1545[tiar-1::s::tev::GFP]) II	CLIP-qPCR
N2	Bristol wild-type	vIPR, CLIP-qPCR, *daz-1* knockdown
EG6699	ttTi5605 II; unc-119(ed3) III; oxEx1578 [eft-3p::GFP + Cbr-unc-119]	MosSCI injections
gld-1 reporter (this study)	[gld-1p::GFP::H2B ORF::gld-1 3'utr]II; unc-119(ed3) III	*daz-1* knockdown
miR-84 site destruction (this study)	*gld-1* 3'UTR edit1 I	vIPR
miR-84 site conversion (this study)	*gld-1* 3'UTR edit2 I	vIPR

Supplementary Table 2. Sequences of vIPR probes

Target	Probe	Sequence	%GC
gfp	1	ccagtgaaaagttcttctcc	45%
	2	tgacagaaaatttgtgccca	40%
	3	ggtagttttccagtagtgca	45%
	4	cgagaagcattgaacaccat	45%
	5	tggcactcttgaaaaagtca	40%
	6	agttcccgtcatctttgaaa	40%
	7	aacaagggtatcaccttcaa	40%
	8	ttgtttgtctgccatgatgt	40%
	9	ccatcttcaatgttgtgtct	40%
	10	cgccaattggagtattttgt	40%
	11	gatctttcgaaagggcagat	45%
	12	agcagctgttacaaactcaa	40%
alg-1	1	aatgaacgtccgacaggagt	50%
	2	cgatactttctgcgcatttg	45%
	3	ttgtcctggaacaatgttgc	45%
	4	catatctgctaggatgagca	45%
	5	gaagaattgcccctcagaaa	45%
	6	tccttgaataccagcatgag	45%
	7	taatatgctggcgctggaat	45%
	8	cagcagtgcatttggacaat	45%
	9	acgtgtggatttgaagtgca	45%
	10	gaggttgagagcgagttttt	45%
gld-1	1	tgtctgttgttcgtcgatag	45%
	2	aacctcgattggttctgtag	45%
	3	aggaagctcaactctcggaa	50%
	4	cataatcttgcatccagtgt	40%
	5	gtcttcgcattgcacaagaa	45%
	6	tacgtaccgttgatgattgc	45%
	7	aagtggttgttgggtacttc	45%
	8	ttggtgtttgtttgattggc	40%
	9	tggcaacatgatgtatggca	45%
	10	taagatgttattaccggggc	45%
lin-41	1	tgctgtggtgactgaatcat	45%
	2	ttggtgtcgaacaatccgac	50%
	3	tgttgcaatggagaccatga	45%
	4	gaagacattggtgttgccaa	45%
	5	tcattttcaacgccagacac	45%
	6	tttccaggatgttgagcaac	45%
	7	ctgtttgacgctcacgaatc	50%
	8	gctgcaatctcatcatagtt	40%
	9	aattgtccatctggcagata	40%
	10	cggatgcaattgtttccgaa	45%

Supplementary Table 3. Sequences of qPCR primers

Primer name	Sequence
18S_f	atggccgttcttagttggtg
18S_r	cgatacctttcggcatagga
alg-1_f	gctcatttggtagcgttccg
alg-1_r	catgacgttgttggcatccg
gapdh_f	tgcaccaccaactgcttagc
gapdh_r	ggcatggactgtggtcatgag
gfp_f	ggccctgtccttttaccagac
gfp_r	tcgtccatgccatgtgtaatc
gld-1_f	tttttccccctctcatctcc
gld-1_r	agcatatggtgaaagcagca
lin-41 CDS_f	gccacaacaacaacagccaa
lin-41 CDS_r	gttgaactgcacggctcatc
lin-41 CDS/3' UTR_f	tgatggtcgcatatacgtcgt
lin-41 CDS/3' UTR_r	ctttgcgcccgatttgatgg
lin-41a 3' UTR (1)_f	tgatcggccccatcaaatcg
lin-41a 3' UTR (1)_r	ccgggtagaagcacaggaaaa
lin-41a 3' UTR (2)_f	tctcccgtactcccaccaat
lin-41a 3' UTR (2)_r	ggggacattaggcaattggga
myo-3_f	cgctgtctctgatgaagcttaccg
myo-3_r	gtacctcccttctttccatccttgg
spn-4_f	ttgtgcagaccaaaccacct
spn-4_r	ccacatcagacagctcgtga
tbb-2_f	gaggccctctacgacatctg
tbb-2_r	aagtgaagacgtgggaatgg

Supplementary Table 4. Sequences of cloning primers

Primer name	Sequence
gld-1 promoter fwd	actACTAGTattgagatacacaagtgtttttta
gld-1 promoter rev	actAGATCTctgtaagaaaacttagtaataatatatttttc
gld-1 3' UTR fwd	actCCTAGGAAAGTTCACATTTATAACTCACACTC
gld-1 3' UTR rev	actCTTAAGTCTCGCCCTGTATTTCAATTTC
gld-1 5' UTR/gfp fwd	actAGATCTGTTAACCATCGAAGAatgagtaaaggagaagaacttttc
gfp rev	actCCTAGGttacttgctggaagtgtacttggtgacg

Supplementary Table 5. Sequences of CRISPR oligonucleotides

CRISPR oligonucleotide	Sequence
seed destruction crRNA	/AltR1/rArG rGrGrG rGrUrG rCrUrU rUrGrG rCrArU rUrCrG rGrUrU rUrUrA rGrArG rCrUrA rUrGrC rU/AltR2/
let-7 conversion crRNA	/AltR1/rGrA rGrGrU rArGrU rArUrG rArArU rGrGrG rUrArG rGrUrU rUrUrA rGrArG rCrUrA rUrGrC rU/AltR2/
*dpy-10*_crRNA	/AltR1/rGrC rUrArC rCrArU rArGrG rCrArC rCrArC rGrArG rGrUrU rUrUrA rGrArG rCrUrA rUrGrC rU/AltR2/
seed destruction ssODN	AACACAGAAAAATAAAGGGGGTGCTTTGGCATTCGTCCAAGTATGAATGGGTAGGGGAATAGCTGCATCAATAAAAGTA
let-7 conversion ssODN	AACACAGAAAAATAAAGGGGGTGCTTTGGCATTCGAGGTAGTAGGATTGGGAAGGGGAATAGCTGCATCAATAAAAGTATCAATGAGTATTCTCC
*dpy-10*_ssODN	CACTTGAACTTCAATACGGCAAGATGAGAATGACTGGAAACCGTACCGCATGCGGTGCCTATGGTAGCGGAGCTTCACATGGCTTCAGACCAACAGCCTAT

References

[1] Rehmsmeier, M., Steffen, P., Hochsmann, M. & Giegerich, R. Fast and effective prediction of microRNA/target duplexes. *RNA* **10**, 1507–1517 (2004).

[2] Broughton, J. P., Lovci, M. T., Huang, J. L., Yeo, G. W. & Pasquinelli, A. E. Pairing beyond the seed supports microRNA targeting specificity. *Mol. Cell* **64**, 320–333 (2016).
