# Supplementary material for: Identification of proteins and miRNAs that specifically bind an mRNA in vivo
Source: Nat Commun. 2019 Sep 16;10:4205. doi: 10.1038/s41467-019-12050-7 (PMC6746756; doi:10.1038/s41467-019-12050-7)
Supplement: Supplementary file 4 — Description of Additional Supplementary Files [file 41467_2019_12050_MOESM4_ESM.docx]

**Description of Additional Supplementary Files**

File name: Supplementary Data 1
Description: RNA sequencing of vIPR samples - kallisto counts and re-normalized TPMs summed up per gene.

File name: Supplementary Data 2
Description: MaxQuant raw data and analysis for vIPR pilot experiment, testing RNA pulldown of *gld-1::gfp* after crosslinking with PFA-XL, PAR-XL or cXL. Reported are peptide numbers and peptide raw intensities for detected proteins in input, supernatant, and pulldown samples (MS_raw). Additionally, proteins enriched against the control in cXL vIPR are listed (enriched_cXL).

File name: Supplementary Data 3
Description: MaxQuant raw data and analysis for cXL vIPR of transgenic and endogenous transcripts. Reported are peptide numbers and LFQ intensities (before and after log_2_-transformation and imputation) for reproducibly detected proteins in pulldown samples. Results from the moderated *t*-test performed on triplicate vIPR experiments are also given.

File name: Supplementary Data 4
Description: GO term analysis for vIPR-identified candidate binders of the *gld-1::gfp* transcript.

File name: Supplementary Data 5
Description: Nanostring nCounter analysis of RNA levels in N2 and *gld-1* 3’ UTR reporter worms after mock and *daz-1* RNAi treatment. Raw counts were normalized to the internal positive controls and to two reference genes (*tbb-1*, *tbb-2*), using the nSolver 4.0 software.

File name: Supplementary Data 6
Description: mirDeep2 miRNA counts from small RNA sequencing of vIPR samples.
