# Supplementary material for: Identification of proteins and miRNAs that specifically bind an mRNA in vivo
Source: Nat Commun. 2019 Sep 16;10:4205. doi: 10.1038/s41467-019-12050-7 (PMC6746756; doi:10.1038/s41467-019-12050-7)
Supplement: Supplementary file 2 — Peer Review [file 41467_2019_12050_MOESM2_ESM.pdf]

Reviewers' comments:

Reviewer #1 (Remarks to the Author):

Summary

In this manuscript Theil et al. describe a method, called vIPR, for the identification of proteins and miRNAs that associate to specific mRNAs in a living organism (C. elegans). Capitalizing on previously described methods, they have optimized protocols based on UV-induced crosslinking of protein/nucleic acids interactions and mRNA pulldown by specific probes. Applying vIPR to genes coding for GFP-tagged proteins, they found RNA-binding proteins and miRNAs that associate with the gld-1 and lin-41 mRNAs.
The concepts underlying the method described by Theil et al (UV-induced crosslink to capture RNA/protein interactions in living cells and specific transcript capture through specific probes) are not new. The prime novelty of this manuscript is their optimization to allow the efficient capture of a moderately expressed mRNA from a living organism and the unbiased identification of co-purified proteins by mass spectrometry. The other aspect of the method, pulldown of specific endogenous transcripts and the subsequent analysis of associated miRNAs, had already been described before (Hassan T et al, NAR 2013).
The isolation and analysis of specific mRNPs has been challenging and the various available approaches come with drawbacks. Hence a robust method to isolate and analyze mRNPs from a living organism is a real advance. However, at this point the general applicability of vIPR and its superiority to existing alternative approaches have not been completely demonstrated. The manuscript may thus be better suited for a more specialized journal.

Main points:

1. The conclusion that vIPR works best with conventional crosslinking (cXL) is drawn from one single example (tiling probes targeting the gfp sequence of gld-1:gfp) and one single MS experiment. It thus appears adventurous to claim that cXL outperforms PFA- and PAR-XL. The crosslinking efficiency obtained from one approach or another probably also depends on which RBP binds to which mRNA, and hence cXL may be optimal for gld-1:gfp but not for another transcript. Since this is a new method that is meant to be adopted by others, it is expected that the authors do more of the "dirty work" and test the claimed superiority of cXL over the two other crosslinking approaches on more transcripts.
2. From what I understand from the main text and method section, RBPs specifically-associated with gld-1:gfp were deduced from comparing the LFQ values of the gld-1:gfp and lin-41:gfp datasets. It does not seem the expression levels of gld-1 and lin-41 (as well as their respective capture efficiencies) were taken into account. This can distort the analysis and lead to false negatives/positives. For example if lin-41 was 10 time less expressed than gld-1 and both bind to the same RBP1 with the same stoichiometry, assuming similar capture efficiencies RBP1 would appear 10 time enriched in the gld-1 dataset and wrongly identified as specific for gld1.
3. In the same vein, different binding stoichiometries may explain different enrichments in one dataset or another. For example, TIAR-2 may have four binding sites on gld-1 but only one on lin-41 and hence be wrongly assigned as a gld1-specific binder.
4. A more balanced discussion on the limitations of vIPR would seem more appropriate. There are probably many more false negative than inferred from the text since the identified specific binders were identified with few peptides only. Considering this and the typical low efficiency of UV-induced crosslinking (1-5% according to Darnell 2010), transient interactions will be probably hard to catch with this technique. This might explain the absence of detected Alg1 from both lin-41 and gld-1 in spite of the miRNA sites. Finally numerous proteins that are associated with and regulate mRNAs do not directly contact RNA (e.g. Ain-1 or motor proteins), these important components of mRNPs will be invisible to vIPR.
5. One potential limitation of the method is its applicability to endogenous transcripts as opposed to

gfp-tagged transgenes. While I agree with the authors that capturing endogenous mRNAs with custom-made specific probes should also work, not working with mRNA coding for gfp-tagged proteins will make the method more challenging. For example:
a. The non target control will require knocking out or heavily mutating the endogenous gene by genome editing. The former is not possible for essential genes whereas the latter may prevent the binding of RNA-binding proteins.
b. The gfp tiling probes do not directly target the transcript of interest and hence are not affected by relevant potential binding sites for interacting RBPs. This will not be the case for probes that are directed against endogenous mRNA sequences. They might thus select for transcripts that do not bind to a given RBP if binding and hybridization sites are overlapping.
c. To identify transcript-specific binders, comparison of interactomes will have to rely on different transcripts captured with distinct sets of probes that may not yield similar capture efficiencies. How to deal with such differences is not addressed in the present manuscript.

At the moment, only applied on tagged mRNAs, the added value of vIPR compared to existing methods such as RaPID (Slobodin & Gerst, RNA, 2010) or Urb-RIP (Cottrell & Djuranovic, PLoS One 2016) is thus questionable. A convincing demonstration of the superiority of vIPR would be its application to untagged endogenous gld-1 and lin-41.

Additional points

1. The paper from Rogell et al, RNA 2017 (Hentze lab) is clearly a precursor of the present manuscript: while it did not reach the ultimate goal of the analysis of a specific mRNP isolated from living cells, it clearly set the stage for a protocol based on probes-mediated isolation of specific transcripts following UV-induced crosslinking, and their subsequent MS analysis. This work should be cited at least in the introduction part.
2. In the discussion section, the discussion on the TRIP approach (1st paragraph) seems unfair for the last point: while it is true that only western blot analysis was performed in the original TRIP paper (ref 38), nothing in principle prevents a user from performing MS analysis on samples obtained from a TRIP experiment. Hence it is not correct to state that TRIP requires a priori knowledge of candidate binders.
3. Fig. 1b. Another choice of color may be chosen for the bars, at the first glance it is hard to distinguish (especially from the shades of gray) what is what.
4. Fig. 4b. I would suggest refraining from presenting data from one single experiment and therefore would recommend either performing the missing LIN-41 CLIP-qPCR experiment for myo-3 or remove myo-3 from this panel.
5. Methods part. Please indicate how long UV irradiation was performed (page 20), which sonification device was used (page 21) and what volume was used to wash the MyOne C1 beads (page 22)
6. Raw RNA-seq and MS data should be made available to public repositories.

Reviewer #2 (Remarks to the Author):

Overview:

This manuscript describes the identification of proteins and RNAs that bind to two C. elegans mRNAs expressed specifically in germline cells. The paper is well written and the figures are nicely prepared and presented. There is no stated hypothesis being tested, but the motivation for pursuing this work is that identifying proteins interacting with RNAs is required in order to understand their regulation and functions in cells.

Extension of the RNA antisense capture technique to C. elegans represents a useful step forward in identifying RNA binding proteins in this organism. It is encouraging that the specific transcript and

associated proteins can be purified specifically from germline cells in the context of the whole organism, and speaks to the power of the hybridization capture methods to identify direct protein interactors.

The comparison between the results of different crosslinking methods is useful, and will help others who are trying to adapt this technique to their organism of interest. Concurrent analysis of proteins and miRNAs binding to the target mRNA transcript also shows the utility of the method and its flexibility in readouts. Overall this is a solid manuscript with valuable contributions to the field.

Specific critiques:

1. The idea to target the gfp portion of the transgene is a good one, since the designed probes can be used to capture other transcripts, as long as those transcripts are fused to the gfp transcript in the worm. One concern would be whether incorporating the gfp into the gene results in loss of particular binding factors or if there are more non-specific binders accumulating on the transcript as a result of including gfp. An important experiment would be to capture the endogenous transcript and show by Western blotting or mass spectrometry that the same identified proteins bind the native transcript in wild-type worms. Please edit the statements in the Introduction and Discussion that the approach "…can be extended to discovering interactions in any other animal or tissue amenable to UV crosslinking", since this would more appropriately be stated as "…amenable to UV crosslinking and genetic methods for creation of stable gfp transgenes".

2. Two capture methods are cited as references for the development of vIPR: ChIRP-MS and RAP-MS. After optimization of crosslinking and elution, the final method is most similar to RAP-MS with the use of UV to create direct RNA-protein crosslinks and benzonase elution to release proteins from captured RNAs. However, a major difference between the two methods is that short 20 nucleotide hybridization probes are used for vIPR, while in RAP-MS the probes are 90 nucleotides in length. The use of shorter probes limits the temperature, salt and other denaturant concentrations that can be used in the wash steps. With short probes, the melting temperature of the DNA:RNA hybrids is too low for the probes to remain stably attached to the target in the highly denaturing washes used for RAP-MS. These high stringency washes are required to remove all nonspecific background proteins and RNAs from the capture beads. The authors note that there are many proteins identified in their "no-target" control and this is likely due to the fact that the washes are not as highly denaturing as in RAP-MS. Please describe this difference in wash stringency and how it might contribute to the observed non-specific background proteins in the control, in the paragraph of the Discussion that begins on page 15, line 356.

3. The introduction frames the limitations of mRNA expression as one of the major problems to be solved with this new approach. The fact that existing methods have been proven to work for to high abundance but not low abundance RNA transcripts is highlighted. However, the implication that the vIPR method reported here is better for "low abundance transcripts" seems slightly misleading and the claims should be softened. The abundance of gld-1 is described as "moderate" in the discussion but in input samples the transcript appears to be in the top ~20 percent of expressed transcripts (i.e. Figure 1C input RNA). Please report the percent of all transcripts above which gld-1 and lin-41 are expressed, to help inform others who might attempt to use this approach about the feasibility of applying vIPR to low abundance transcripts. In addition, on page 15 there is a statement that gld-1 and lin-41 are expressed at much lower levels than Xist. The current understanding is that there are 50-100 copies of Xist in each cell, based on high resolution single-cell microscopy experiments (Sunwoo, Wu, and Lee, PNAS 2015 doi: 10.1073/pnas.1503690112). Please provide a citation to this article and remove the statement that gld-1 is at least ten times less abundant than Xist.

4. Another aspect that is highlighted as a weakness of previous studies is the quantity of input material required for captures. The material quantity used here was similar to other methods but only a single peptide was required for protein identifications in this study, as opposed to other methods

that generally use at least two unique peptides to assign protein identities. These results suggest that the quantity of recovered protein from each capture was low. Please specify whether the single peptides used to identify each protein are unique peptides, or whether they could potentially be assigned to multiple proteins. The multiple replicates performed for optimized vIPR captures and the subsequent validation of a few interacting factors does lend support to the idea that at least some of these protein identifications are correct. However, it would still be helpful to others trying to perform similar experiments if a description of the total quantity of protein recovered and analyzed by mass spectrometry for each sample can be provided.

5. Please precisely define the identity of the "no-target" control. Is it a randomly scrambled oligonucleotide sequence? Is it a specific biological sequence that does not exist in C. elegans but does exist in other species? Or is it a transcript that is in encoded in the C. elegans genome but is not expressed in these particular worms? Please provide the sequences of capture probes for no-target control and explain the details of how the control probes were designed.

6. There is another article describing a similar approach for identifying proteins interacting with viral RNAs that should also be cited in the Introduction: Phillips, Garcia-Blanco and Bradrick, Methods 2014. https://doi.org/10.1016/j.ymeth.2015.08.008

7. Please define the acronym "FBF proteins" on page 5, line 115.

8. For all RNA sequencing data, please deposit the raw data to NCBI Gene Expression Omnibus and provide accession numbers.

9. For all proteomics data, please deposit the raw data output files in the original instrument vendor file format to PRIDE Archive or a similar repository that is part of the ProteomeXchange Consortium and provide accession numbers.

Reviewer #3 (Remarks to the Author):

The manuscript by Dr. Rajewsky and colleagues describes a novel approach to identification of proteins and small RNAs that form a complex with an mRNA of interest in vivo. This approach overcomes low abundance of endogenously expressed mRNAs and is powerful enough to analyze mRNAs with tissue-specific expression, which would be of high general interest. The vIPR approach recovered the known RNA-binding proteins (RBPs) in complex with gld-1 mRNA and identified new RBPs that bind gld-1 and lin-41 mRNAs. The new RBP/mRNA interactions were confirmed by an independent approach, and the new miRNA/mRNA interactions were validated by the analysis of previously reported small RNA sequencing datasets. These are significant findings that advance the field's capabilities for studying post-transcriptional regulation of gene expression. However, there are some concerns regarding the study that need to be addressed. These are outlined below:

Major points:
1. The vIPR analysis recovered a novel association of DAZ-1 RBP with gld-1 mRNA, but the study does not provide an indication whether this specific association reflects any regulatory role for DAZ-1 in controlling gld-1 expression. The manuscript would be significantly strengthened if there were any indications that DAZ-1 is functionally involved in gld-1 regulation.

2. Relevant to the analysis in Fig. 3a and in Supplementary Fig. 3d: It appears that the statistical significance of protein enrichment with the isolated mRNAs was calculated with a Student's 2-tailed t-

test for hundreds of individual proteins without a correction for multiple comparisons. The P value statistic needs to be corrected for false-discovery rate, for example through the Benjamini-Hochberg procedure (to account for incorrect assignment of significance).

3. The authors emphasize that vIPR recovers endogenous in vivo tissue-specific interactions (eg in line 339), but this statement needs to be reevaluated in regards to the recovered miRNAs. Expression of lin-4 might not be exclusive for germline as it is expressed in the hypodermis during larval development. In fact, the hypodermis is the site of lin-41 regulation by let-7 miRNA. Furthermore, germline expression of LIN-41 is not controlled by let-7 (Spike et al., 2014), so what is the relevance of recovered let-7 mRNA to lin-41 regulation? Does it come from the hypodermis, in which case the recovered complex is not germline-specific? Does it come from the germline reflecting some non-functional interaction? There are similar concerns regarding miRNAs associated with gld-1 mRNA, as none of them appear germline-enriched (McEwen et al., 2016), so does the enrichment in the pulldown procedure reflect in vivo regulation?

Minor points:
1. Fig. 1 Legend. What is meant by "stringent lysis" (panel A)? Only a single lysis protocol is described.

2. The comparison of proteins enriched with gld-1 and lin-41 mRNAs was helpful in identifying specific regulators. However, the logic discarding all common regulators as "promiscuous binders without regulatory impact" (lines 365-367) is faulty: both gld-1 and lin-41 mRNAs are non-uniformly expressed in the germlines, and might in fact be regulated by same RBPs (eg to repress their expression in the stem cells). If the authors wanted to select against RBPs that do not have regulatory impact, they might be served better to choose a housekeeping gene that is uniformly expressed in the germline (such as GFP::Tubulin fusion).

RESPONSE TO REFEREES

We thank the reviewers for their critical and constructive comments on our manuscript.

The reviewers agreed that the "Extension of the RNA antisense capture technique to *C. elegans* represents a useful step forward in identifying RNA binding proteins in this organism", and that a method to analyze endogenous mRNPs is of "high general interest"/"a real advance." Furthermore, they found that the manuscript is solid with "valuable contributions to the field"/"significant findings that advance the field's capabilities for studying post-transcriptional regulation of gene expression."

The reviewers' main criticism was that we performed experiments with *gfp*-tagged mRNAs, and did not provide evidence that the method also works for endogenous transcripts. They stated that "A convincing demonstration of the superiority of vIPR would be its application to untagged endogenous gld-1 and lin-41" /"An important experiment would be to capture the endogenous transcript and show by Western blotting or mass spectrometry that the same identified proteins bind the native transcript in wild-type worms."

We successfully performed the requested vIPR experiments with endogenous *gld-1* and *lin-41*, demonstrating a high overlap in identified candidate binders from pulldowns with *gfp*-tagged and endogenous transcripts.

Concerning the biological relevance of our findings, it was commented that "The manuscript would be significantly strengthened if there were any indications that DAZ-1 is functionally involved in gld-1 regulation."

To address this point, we generated a *gld-1 in vivo* reporter and show that both transcript and protein levels are downregulated upon *daz-1* knockdown, supporting the suggested role of DAZ-1 in transcript stabilization and translational activation.

Additionally, we were asked to address the tissue specificity and biological role of the miRNA-transcript interactions that we identify.

While we did not unravel tissue-specific regulation, we provide additional evidence that vIPR can be used to recover biologically important interactions by performing vIPR experiments with the *alg-1* transcript, which led to enrichment of the known *alg-1* regulator *miR-71*. Furthermore, we validated the predicted *miR-84* binding site in the *gld-1* 3' UTR by loss-of-function and gain-of-function (change of specificity) mutations via CRISPR-editing and subsequent *gld-1* pulldowns.

Finally, we were asked to more extensively discuss the limitations of vIPR (required transcript levels, missed binders, protein background) and tone down statements concerning the superiority of cXL crosslinking.

We changed the text accordingly.

Altogether, we believe that the revision improved our manuscript substantially and are confident that it is now ready for publication.

Point-by-point response:

Reviewer #1 (Remarks to the Author):

Summary

In this manuscript Theil et al. describe a method, called vIPR, for the identification of proteins and miRNAs that associate to specific mRNAs in a living organism (C. elegans). Capitalizing on previously described methods, they have optimized protocols based on UV-induced crosslinking of protein/nucleic acids interactions and mRNA pulldown by specific probes. Applying vIPR to genes coding for GFP-tagged proteins, they found RNA-binding proteins and miRNAs that associate with the gld-1 and lin-41 mRNAs.

The concepts underlying the method described by Theil et al (UV-induced crosslink to capture RNA/protein interactions in living cells and specific transcript capture through specific probes) are not new. The prime novelty of this manuscript is their optimization to allow the efficient capture of a moderately expressed mRNA from a living organism and the unbiased identification of co-purified proteins by mass spectrometry. The other aspect of the method, pulldown of specific endogenous transcripts and the subsequent analysis of associated miRNAs, had already been described before (Hassan T et al, NAR 2013).

>> We added the citation to the corresponding results section. The procedure differs from vIPR in that it captures transcripts by single probes which are designed based on *in silico* secondary structure predictions. The transcript enrichments reported are between 16- and 75-fold against control RNAs (vIPR enables enrichment of ~20,000-fold against a control RNA), capture efficiencies are not reported.

The isolation and analysis of specific mRNPs has been challenging and the various available approaches come with drawbacks. Hence a robust method to isolate and analyze mRNPs from a living organism is a real advance. However, at this point the general applicability of vIPR and its superiority to existing alternative approaches have not been completely demonstrated. The manuscript may thus be better suited for a more specialized journal.

Main points:

1. The conclusion that vIPR works best with conventional crosslinking (cXL) is drawn from one single example (tiling probes targeting the gfp sequence of gld-1:gfp) and one single MS experiment. It thus appears adventurous to claim that cXL outperforms PFA- and PAR-XL. The crosslinking efficiency obtained from one approach or another probably also depends on which RBP binds to which mRNA, and hence cXL may be optimal for gld-1:gfp but not for another transcript. Since this is a new method that is meant to be adopted by others, it is expected that the authors do more of the “dirty work” and test the claimed superiority of cXL over the two other crosslinking approaches on more transcripts.

>> The main focus of our study was not to compare different crosslinking methods comprehensively, which is not a straightforward task, but to develop a pulldown strategy that allows retrieval of direct *in vivo* RNA binders from *C. elegans*. We rephrased the corresponding parts of the manuscript, stressing that this was a pilot experiment, and pointed out the differences between the tested crosslinking methods as well as biases and drawbacks of each method.

2. From what I understand from the main text and method section, RBPs specifically-associated with gld-1:gfp were deduced from comparing the LFQ values of the gld-1:gfp and lin-41:gfp datasets. It does not seem the expression levels of gld-1 and lin-41 (as well as their respective capture efficiencies) were taken into account. This can distort the analysis and lead to false negatives/positives. For example if lin-41 was 10 time less expressed than gld-1 and both bind to the same RBP1 with the same stoichiometry, assuming similar capture efficiencies RBP1 would appear 10 time enriched in the gld-1 dataset and wrongly identified as specific for gld1.

>> We thank the reviewer for this comment and agree that expression levels should be taken into account. Capture efficiencies were similar for all tested transcripts (between 60-80%; **Figure 1b**, **Supplementary Fig. 1h** and **Supplementary Fig. 4c**). To assess target expression, we performed RNA sequencing on pulldown input samples, and found that the *gld-1::gfp* transcript was ~1.7x more highly expressed than the *gfp::lin-41* transcript (**Supplementary Fig. 1d,e**). We changed our analysis in **Fig. 4a** to factor this difference in.

On a general note, since both crosslinking and mass spectrometry are inherently variable, the comparison of enrichment only represents a means to facilitate identification of candidates for specific regulation. These candidates will always need to be validated by independent methods (*e.g.*, CLIP).

3. In the same vein, different binding stoichiometries may explain different enrichments in one dataset or another. For example, TIAR-2 may have four binding sites on gld-1 but only one on lin-41 and hence be wrongly assigned as a gld1-specific binder.

>> The comparison of vIPR of two transcripts allows identification of differential binding. Following up on the given example, we believe that it is reasonable to assume that a transcript bound at four sites is under stronger regulatory control than a transcript bound at one site. However, we agree that many transcripts would need to be assessed to evaluate the specificity of an RBP. We addressed this by performing CLIP experiments. We agree with the reviewer that depending on the chosen transcripts, RBPs specific to both transcripts might be mis-classified. In general, vIPR and similar methods provide lists of candidates that, of course, need to be validated independently. We changed the corresponding results and discussion section to make this clearer.

4. A more balanced discussion on the limitations of vIPR would seem more appropriate. There are probably many more false negative than inferred from the text since the identified specific binders were identified with few peptides only. Considering this and the typical low efficiency of UV-induced crosslinking (1-5% according to Darnell 2010), transient interactions will be probably hard to catch with this technique. This might explain the absence of detected Alg1 from both lin-41 and gld-1 in spite of the miRNA sites. Finally numerous proteins that are associated with and regulate mRNAs do not directly contact RNA (e.g. Ain-1 or motor proteins), these important components of mRNPs will be invisible to vIPR.

>> We edited the text to discuss the limitations of vIPR more extensively. It is true that we likely miss binders, especially dsRBPs, or RBPs that bind to sites that do not favor crosslinking. We also discuss the fact that we do not find ALG-1 in any of our data sets, and we point out, that our method is designed to identify binders that directly associate with the mRNAs under study. To identify mRNP

components that bind indirectly by complexing with RBPs, complementary approaches will need to be taken.

5. One potential limitation of the method is its applicability to endogenous transcripts as opposed to gfp-tagged transgenes. While I agree with the authors that capturing endogenous mRNAs with custom-made specific probes should also work, not working with mRNA coding for gfp-tagged proteins will make the method more challenging. For example:
a. The non target control will require knocking out or heavily mutating the endogenous gene by genome editing. The former is not possible for essential genes whereas the latter may prevent the binding of RNA-binding proteins.

>> We agree that it is less straight-forward to control for unspecific background when assessing endogenous transcripts. As the reviewer stated, knocking out or editing probe binding sites within an endogenous transcript is not feasible, since this might lead to destruction/generation of RBP binding sites and/or secondary effects. We tested pulldown of three endogenous transcripts (*gld-1*, *lin-41*, and *alg-1*) and used the same control as for the *gfp*-tagged transcripts (pulldown with *gfp*-complementary probes in the wild-type strain). Although this control does not account for probe-specific background, there is a high overlap of factors binding to *gfp*-tagged and endogenous *gld-1* and *lin-41* (**Fig. 3**, **Fig. 4**, **Supplementary Fig. 4**).

b. The gfp tiling probes do not directly target the transcript of interest and hence are not affected by relevant potential binding sites for interacting RBPs. This will not be the case for probes that are directed against endogenous mRNA sequences. They might thus select for transcripts that do not bind to a given RBP if binding and hybridization sites are overlapping.

>> We captured the *gfp*-tagged transcripts with probes only targeting the *gfp* CDS, while using probes covering both CDS and 3' UTR for the endogenous transcripts. As discussed above, overlap of enriched proteins in vIPR of *gfp*-tagged and endogenous transcripts was high in our experiments. Importantly, as the reviewer stated in 4., the crosslinking efficiency of UV 254 nm is low. Thus, it is not expected that many proteins will simultaneously crosslink to the same transcript. Since we use 10-12 probes per transcript, it will be efficiently retrieved even if one or few probe binding sites will not be accessible due to linkage to a crosslinked protein.

c. To identify transcript-specific binders, comparison of interactomes will have to rely on different transcripts captured with distinct sets of probes that may not yield similar capture efficiencies. How to deal with such differences is not addressed in the present manuscript.

>> We measured capture efficiencies for both transgenic and endogenous transcripts, in total using 4 sets of probes for 5 transcripts. We observed similar capture efficiencies for all transcripts.

At the moment, only applied on tagged mRNAs, the added value of vIPR compared to existing methods such as RaPID (Slobodin & Gerst, RNA, 2010) or Urb-RIP (Cottrell & Djuranovic, PLoS One 2016) is thus questionable. A convincing demonstration of the superiority of vIPR would be its application to untagged endogenous gld-1 and lin-41.

>> RaPID and Urb-RIP require co-expression of two constructs: an aptamer-tagged target transcript and an aptamer binding protein (whose expression has to be tightly controlled in the case of RaPID). Both methods and variants thereof were, to our knowledge, so far only tested in cell culture (mammalian cells, yeast, bacteria), and oftentimes rely on overexpression by transfection. They are thus not easily transferable to the worm. The use of *gfp*-complementary probes in vIPR allows straight-forward application of the method to any of the hundreds of available *C. elegans* strains expressing *gfp*-fusion transcripts. With the advent of CRISPR technologies, it also became possible to readily introduce heterologous sequences into endogenous loci, enabling insertion of the *gfp* sequence into genes of interests.

To test whether our method also works for endogenous transcripts, we performed the requested vIPR experiments of untagged, endogenous *gld-1* and *lin-41*.

Additional points

1. The paper from Rogell et al, RNA 2017 (Hentze lab) is clearly a precursor of the present manuscript: while it did not reach the ultimate goal of the analysis of a specific mRNP isolated from living cells, it clearly set the stage for a protocol based on probes-mediated isolation of specific transcripts following UV-induced crosslinking, and their subsequent MS analysis. This work should be cited at least in the introduction part.

>> We added the citation to our introduction and discussion. However, the notion that this study "set the stage for a protocol based on probes-mediated isolation of specific transcripts following UV-induced crosslinking, and their subsequent MS analysis" is misleading. The studies by, *e.g.*, McHugh *et al*. (2015, Nature) and Minajigi *et al*. (2015, Science) established protocols for this before and are introduced and discussed in our manuscript.

2. In the discussion section, the discussion on the TRIP approach (1st paragraph) seems unfair for the last point: while it is true that only western blot analysis was performed in the original TRIP paper (ref 38), nothing in principle prevents a user from performing MS analysis on samples obtained from a TRIP experiment. Hence it is not correct to state that TRIP requires a priori knowledge of candidate binders.

>> We removed the statement. However, we want to point out that a protein detected by western blot will not necessarily be identified by mass spectrometry. Detection by mass spectrometry highly depends on the dynamic range of proteins within the sample. That is, if there is a high background of bead- or probe-binding proteins, transcript-specific proteins might be missed. This has not been assessed by Matia-González *et al*. (2017, Methods).

3. Fig. 1b. Another choice of color may be chosen for the bars, at the first glance it is hard to distinguish (especially from the shades of gray) what is what.

>> We changed the colors.

4. Fig. 4b. I would suggest refraining from presenting data from one single experiment and therefore would recommend either performing the missing LIN-41 CLIP-qPCR experiment for myo-3 or remove myo-3 from this panel.

>> We did the additional measurement and added the data to the chart.

5. Methods part. Please indicate how long UV irradiation was performed (page 20), which sonification device was used (page 21) and what volume was used to wash the MyOne C1 beads (page 22)

>> We added the necessary information.

6. Raw RNA-seq and MS data should be made available to public repositories.

>> We deposited the raw RNA-seq and MS data in public repositories:

RNA-seq:
To review GEO accession GSE130733:
Go to https://www.ncbi.nlm.nih.gov/geo/query/acc.cgi?acc=GSE130733
Enter token ozozmckwhpihhgj into the box

MS:
The mass spectrometry proteomics data have been deposited to the ProteomeXchange Consortium via the PRIDE partner repository with the dataset identifier PXD013720
Website: http://www.ebi.ac.uk/pride
Reviewer account details:
Username: reviewer67738@ebi.ac.uk
Password: 9edfneUB

Reviewer #2 (Remarks to the Author):

Overview:
This manuscript describes the identification of proteins and RNAs that bind to two C. elegans mRNAs expressed specifically in germline cells. The paper is well written and the figures are nicely prepared and presented. There is no stated hypothesis being tested, but the motivation for pursuing this work is that identifying proteins interacting with RNAs is required in order to understand their regulation and functions in cells. Extension of the RNA antisense capture technique to C. elegans represents a useful step forward in identifying RNA binding proteins in this organism. It is encouraging that the specific transcript and associated proteins can be purified specifically from germline cells in the context of the whole organism, and speaks to the power of the hybridization capture methods to identify direct protein interactors. The comparison between the results of different crosslinking methods is useful, and will help others who are trying to adapt this technique to their organism of interest. Concurrent analysis of proteins and miRNAs binding to the target mRNA transcript also shows the utility of the method and its flexibility in readouts. Overall this is a solid manuscript with valuable contributions to the field.

Specific critiques:
1. The idea to target the gfp portion of the transgene is a good one, since the designed probes can be used to capture other transcripts, as long as those transcripts are fused to the gfp transcript in the worm. One concern would be whether incorporating the gfp into the gene results in loss of

particular binding factors or if there are more non-specific binders accumulating on the transcript as a result of including gfp. An important experiment would be to capture the endogenous transcript and show by Western blotting or mass spectrometry that the same identified proteins bind the native transcript in wild-type worms. Please edit the statements in the Introduction and Discussion that the approach "…can be extended to discovering interactions in any other animal or tissue amenable to UV crosslinking", since this would more appropriately be stated as "…amenable to UV crosslinking and genetic methods for creation of stable gfp transgenes".

>> We performed the requested vIPR experiments for the untagged endogenous transcripts, and demonstrate a high overlap of the identified binders. Therefore, we left the quoted statement in its original form.

2. Two capture methods are cited as references for the development of vIPR: ChIRP-MS and RAP-MS. After optimization of crosslinking and elution, the final method is most similar to RAP-MS with the use of UV to create direct RNA-protein crosslinks and benzonase elution to release proteins from captured RNAs. However, a major difference between the two methods is that short 20 nucleotide hybridization probes are used for vIPR, while in RAP-MS the probes are 90 nucleotides in length. The use of shorter probes limits the temperature, salt and other denaturant concentrations that can be used in the wash steps. With short probes, the melting temperature of the DNA:RNA hybrids is too low for the probes to remain stably attached to the target in the highly denaturing washes used for RAP-MS. These high stringency washes are required to remove all nonspecific background proteins and RNAs from the capture beads. The authors note that there are many proteins identified in their "no-target" control and this is likely due to the fact that the washes are not as highly denaturing as in RAP-MS. Please describe this difference in wash stringency and how it might contribute to the observed non-specific background proteins in the control, in the paragraph of the Discussion that begins on page 15, line 356.

>> We included the requested discussion of the potentially higher protein background with vIPR. Of note, we find similar capture efficiencies as well as similar enrichments of our target transcripts compared to the Xist transcript pulldown by RAP-MS (McHugh *et al*. 2015, Nature; Extended Data Fig. 1a,b). This suggests that the RNA background is similar with both methods. As reviewer #1 pointed out, probe binding might interfere with crosslinked proteins. Using shorter probes, as in vIPR, could be of advantage as the risk of an overlap of a crosslinked site with a probe binding site increases with probe length.

3. The introduction frames the limitations of mRNA expression as one of the major problems to be solved with this new approach. The fact that existing methods have been proven to work for to high abundance but not low abundance RNA transcripts is highlighted. However, the implication that the vIPR method reported here is better for "low abundance transcripts" seems slightly misleading and the claims should be softened. The abundance of gld-1 is described as "moderate" in the discussion but in input samples the transcript appears to be in the top ~20 percent of expressed transcripts (i.e. Figure 1C input RNA). Please report the percent of all transcripts above which gld-1 and lin-41 are expressed, to help inform others who might attempt to use this approach about the feasibility of applying vIPR to low abundance transcripts. In addition, on page 15 there is a statement that gld-1 and lin-41 are expressed at much lower levels than Xist. The current understanding is that there are 50-100 copies of Xist in each cell, based on high resolution single-cell microscopy

experiments (Sunwoo, Wu, and Lee, PNAS 2015 doi: 10.1073/pnas.1503690112). Please provide a citation to this article and remove the statement that gld-1 is at least ten times less abundant than Xist.

>> We performed RNA sequencing to determine relative transcript abundances of our target transcripts. We now report TPM distributions of all protein-coding transcripts (**Supplementary Fig. 1d,e,g**). Both *gld-1::gfp* (250 TPM) and gfp::*lin-41* (147 TPM) are amongst the top 15% of expressed transcripts. While experiments were successful with both transgenic and endogenous *gld-1* and *lin-41*, we did not identify significantly enriched proteins for the endogenous *alg-1* transcript (22 TPM), which is amongst the bottom 25% of expressed transcripts. We changed the text to make the reader aware of the limitation that the method, in its current state, will not allow reliable identification of binders for lowly expressed transcripts.
Several different cell types were used for Xist pulldown (Chu *et al.* 2015, Cell; McHugh *et al.* 2015, Nature; Minajigi *et al*. 2015, Science), and reported copy numbers vary depending on cell type and differentiation state. The study by Sunwoo *et al*. (2015, PNAS) estimates 50-100 copies/cell in MEF cells, while in differentiating mouse ES cells, the Xist copy number was estimated to be 300 transcripts/cell (Sun *et al*. 2006, Mol Cell). The male ES cells with inducible Xist that were used for the RAP-MS experiments, were reported to express ~12 times more Xist than female differentiating ES cells (McHugh *et al*. 2015, Nature). Buzin *et al*. (1994, Development) reported levels of < 2,000 copies/cell in mouse adult kidney and embryos. We agree that no comparison should be made and removed the statement.

4. Another aspect that is highlighted as a weakness of previous studies is the quantity of input material required for captures. The material quantity used here was similar to other methods but only a single peptide was required for protein identifications in this study, as opposed to other methods that generally use at least two unique peptides to assign protein identities. These results suggest that the quantity of recovered protein from each capture was low. Please specify whether the single peptides used to identify each protein are unique peptides, or whether they could potentially be assigned to multiple proteins. The multiple replicates performed for optimized vIPR captures and the subsequent validation of a few interacting factors does lend support to the idea that at least some of these protein identifications are correct. However, it would still be helpful to others trying to perform similar experiments if a description of the total quantity of protein recovered and analyzed by mass spectrometry for each sample can be provided.

>> We did not mean to stress the amount of input as a general weakness of previous studies. We removed ambiguous phrasing.
To be considered, we required a protein to be quantified in all three target pulldown samples measured together. Quantification was done based on razor and unique peptides, as is the standard setting in MaxQuant. Razor peptides are ambiguous peptides that are assigned to the protein more likely to be present in the sample, based on the number of additional unique peptides.
Most of our identified candidate binders shown in **Figure 3** and **Supplementary Figure 4** are identified with more than one unique peptide and are assigned unambiguously. We added the numbers of peptides, razor+unique peptides, and unique peptides for the detected protein groups in the corresponding supplementary tables. We additionally changed the table in **Figure 2e** to report unique peptides per protein.

While the protein groups reported by MaxQuant are in the vast majority of cases made up of different isoforms of the same protein, in few cases, these groups contain proteins expressed from different genes, sharing high sequence similarity. For instance, in our pilot experiment, FBF-2 and FBF-1 are grouped together. This is because there were two peptides identified that are unique to FBF-2 and one peptide that could derive from presence of both FBF-2 and FBF-1. In these cases, the conservative assumption is taken that only the protein with the uniquely identified peptides is present in the sample. These specifics can be looked up for proteins of interest to the reader in the peptides tables that we deposited together with the raw data in the PRIDE database.
As reviewer #1 pointed out, protein enrichment also depends on stoichiometry of the complexes. Xist is longer (17 kb in mouse) than most *C. elegans* mRNAs (our assessed transcripts - *gld-1*: 2.1 kb, *lin-41*: 4.3 kb, *alg-1*: 4.8 kb) and contains several tandem repeat sequences, that are likely bound by several copies of a protein. Thus, numbers of identified peptides cannot readily be used as a proxy to compare vIPR with the cited cell culture RNA pulldown methods. We toned down respective statements in our discussion.
To get a rough estimate of protein quantities, we calculated the percentages of Trypsin and Benzonase of the total peptide intensities in a sample and compared these percentages between pulldown input (known quantity) and elution samples. Based on this, we estimate the protein amount in pulldown samples to be ~250 ng. We added this to the corresponding methods section.

5. Please precisely define the identity of the “no-target” control. Is it a randomly scrambled oligonucleotide sequence? Is it a specific biological sequence that does not exist in C. elegans but does exist in other species? Or is it a transcript that is in encoded in the C. elegans genome but is not expressed in these particular worms? Please provide the sequences of capture probes for no-target control and explain the details of how the control probes were designed.

>> This is a misunderstanding. Our no-target control is a pulldown experiment with the same *gfp*-complementary probes, but applied in the wild-type N2 *C. elegans* strain (which does not express *gfp* transcripts). We edited the text to make this clearer.

6. There is another article describing a similar approach for identifying proteins interacting with viral RNAs that should also be cited in the Introduction: Phillips, Garcia-Blanco and Bradrick, Methods 2014. https://doi.org/10.1016/j.ymeth.2015.08.008

>> We added the citation.

7. Please define the acronym “FBF proteins” on page 5, line 115.

>> Done.

8. For all RNA sequencing data, please deposit the raw data to NCBI Gene Expression Omnibus and provide accession numbers.

>> The RNA sequencing data is available for review under the accession number GSE130733:
Go to https://www.ncbi.nlm.nih.gov/geo/query/acc.cgi?acc=GSE130733
Enter token ozozmckwhpihhgj into the box

9. For all proteomics data, please deposit the raw data output files in the original instrument vendor file format to PRIDE Archive or a similar repository that is part of the ProteomeXchange Consortium and provide accession numbers.

>> The mass spectrometry proteomics data have been deposited to the ProteomeXchange Consortium via the PRIDE partner repository with the dataset identifier PXD013720.

Website: http://www.ebi.ac.uk/pride
Reviewer account details:
Username: reviewer67738@ebi.ac.uk
Password: 9edfneUB

Reviewer #3 (Remarks to the Author):
The manuscript by Dr. Rajewsky and colleagues describes a novel approach to identification of proteins and small RNAs that form a complex with an mRNA of interest in vivo. This approach overcomes low abundance of endogenously expressed mRNAs and is powerful enough to analyze mRNAs with tissue-specific expression, which would be of high general interest. The vIPR approach recovered the known RNA-binding proteins (RBPs) in complex with gld-1 mRNA and identified new RBPs that bind gld-1 and lin-41 mRNAs. The new RBP/mRNA interactions were confirmed by an independent approach, and the new miRNA/mRNA interactions were validated by the analysis of previously reported small RNA sequencing datasets. These are significant findings that advance the field's capabilities for studying post-transcriptional regulation of gene expression. However, there are some concerns regarding the study that need to be addressed. These are outlined below:

Major points:
1. The vIPR analysis recovered a novel association of DAZ-1 RBP with gld-1 mRNA, but the study does not provide an indication whether this specific association reflects any regulatory role for DAZ-1 in controlling gld-1 expression. The manuscript would be significantly strengthened if there were any indications that DAZ-1 is functionally involved in gld-1 regulation.

>> We performed RNAi knockdown experiments of *daz-1* and assessed RNA levels of endogenous *gld-1* as well as RNA and protein levels of a *gld-1* reporter (**Fig. 4c,d,e**). Both RNA and protein levels of *gld-1* are reduced upon *daz-1* knockdown supporting a role of DAZ-1 in RNA stabilization and translation.

2. Relevant to the analysis in Fig. 3a and in Supplementary Fig. 3d: It appears that the statistical significance of protein enrichment with the isolated mRNAs was calculated with a Student's 2-tailed t-test for hundreds of individual proteins without a correction for multiple comparisons. The P value statistic needs to be corrected for false-discovery rate, for example through the Benjamini-Hochberg procedure (to account for incorrect assignment of significance).

>> We re-analyzed our data, now using a moderated *t*-test, implemented in the Bioconductor limma package (Smyth 2004, Stat Appl Genet Mol Biol). To correct for multiple comparisons and estimate false discovery rate, we applied the Benjamini-Hochberg procedure.

3. The authors emphasize that vIPR recovers endogenous in vivo tissue-specific interactions (eg in line 339), but this statement needs to be reevaluated in regards to the recovered miRNAs. Expression of lin-41 might not be exclusive for germline as it is expressed in the hypodermis during larval development. In fact, the hypodermis is the site of lin-41 regulation by let-7 miRNA. Furthermore, germline expression of LIN-41 is not controlled by let-7 (Spike et al., 2014), so what is the relevance of recovered let-7 mRNA to lin-41 regulation? Does it come from the hypodermis, in which case the recovered complex is not germline-specific? Does it come from the germline reflecting some non-functional interaction? There are similar concerns regarding miRNAs associated with gld-1 mRNA, as none of them appear germline-enriched (McEwen et al., 2016), so does the enrichment in the pulldown procedure reflect in vivo regulation?

>> It is true that expressing the *lin-41* transcript from its endogenous promoter, we cannot distinguish whether the observed interaction between *lin-41* and *let-7* is recovered from somatic tissue, or whether it represents a non-functional association in the germline. We now made that clearer in the text. Since *gld-1* is only expressed in the germline, we assume the interaction with *miR-84* to occur there.
While we believe that unraveling tissue-specific miRNA regulation is beyond the scope of this manuscript, we 1) provide evidence that vIPR can recover biologically important interactions (in addition to the *lin-41::let-7* interaction, we identified the known interaction of *alg-1* with *miR-71*), and 2) validated the predicted *miR-84* binding site in the *gld-1* 3’ UTR by CRISPR-editing of the endogenous *gld-1* 3’ UTR with subsequent *gld-1* pulldowns (**Fig. 5**).

Minor points:
1. Fig. 1 Legend. What is meant by "stringent lysis" (panel A)? Only a single lysis protocol is described.

>> We removed the word “stringent”.

2. The comparison of proteins enriched with gld-1 and lin-41 mRNAs was helpful in identifying specific regulators. However, the logic discarding all common regulators as "promiscuous binders without regulatory impact" (lines 365-367) is faulty: both gld-1 and lin-41 mRNAs are non-uniformly expressed in the germlines, and might in fact be regulated by same RBPs (eg to repress their expression in the stem cells). If the authors wanted to select against RBPs that do not have regulatory impact, they might be served better to choose a housekeeping gene that is uniformly expressed in the germline (such as GFP::Tubulin fusion).

>> We agree with the reviewer and changed the text accordingly.

REVIEWERS' COMMENTS:

Reviewer #1 (Remarks to the Author):

Theil et al. did a great job in updating their manuscript. The rebuttal letter, new data and updated discussion adequately address the points I had raised on their original manuscript. I can therefore enthusiastically recommend it for publication.

Reviewer #2 (Remarks to the Author):

The authors have addressed my comments in a satisfactory manner.

Reviewer #3 (Remarks to the Author):

The manuscript by Theil et al has been significantly expanded and reworked in response to reviewers' comments. Virtually all of the reviewers' concerns have been addressed. Significant improvements include: performing vIPR on the endogenous untagged transcripts and the finding that targeting the GFP-tagged transcript identifies a similar protein binder set as targeting of the endogenous transcript. Validating functional interaction of DAZ-1 with gld-1 mRNA further underscores the utility of vIPR. Finally, mutational analysis of predicted miRNA interaction site in gld-1 3'UTR authenticates the specificity of recovered interactions. The manuscript is now ready for the publication.
